# Genome Annotation of Molting-Related Protein-Coding Genes in *Propsilocerus akamusi* Reveals Transcriptomic Responses to Heavy Metal Contamination

**DOI:** 10.3390/insects16060636

**Published:** 2025-06-17

**Authors:** Wenbin Liu, Anmo Zhou, Ziming Shao, Jiaxin Nie, Chuncai Yan, Shaobo Gao, Yiwen Wang

**Affiliations:** 1 College of Life Sciences, Tianjin Normal University, Tianjin 300387, China; skylwb@tjnu.edu.cn (W.L.); skyzam@stu.tjnu.edu.cn (A.Z.); 2410170020@stu.tjnu.edu.cn (Z.S.); 2410170017@stu.tjnu.edu.cn (J.N.); 2 Grassland Research Institute, Chinese Academy of Agricultural Sciences, Hohhot 010010, China; 3 School of Pharmaceutical Science and Technology, Tianjin University, Tianjin 300072, China; 4 Shanxi Key Laboratory of Nucleic Acid Biopesticides, Shanxi University, Taiyuan 237016, China

**Keywords:** *Propsilocerus akamusi*, juvenile hormone, ecdysone, chitin, metal transport, stress

## Abstract

The exoskeleton of insects, known as the cuticle, necessitates regular renewal during molting and metamorphosis, with chitin being its primary structural component. Consequently, the molting and metamorphosis processes in insects are characterized by periodic degradation and synthesis of chitin, which are tightly regulated by juvenile hormone (JH) and 20-hydroxyecdysone (20E). *Propsilocerus akamusi*, a species that plays a crucial role in freshwater ecosystems, demonstrates remarkable resilience to environmental pollutants, including metallic elements found in industrial waste. In this investigation, we systematically analyzed and summarized the metabolic pathways associated with JH, 20E, chitin, and heavy metal transport in *P. akamusi*. Based on previous genome assembly, we conducted a systematic annotation and functional analysis of genes involved in these metabolic pathways in *P*. *akamusi*. This was achieved by querying conserved domains using Pfam and SMART, as well as identifying gene-specific classical consensus regions through amino acid sequence alignment using DNAMAN. Through our validation, a total of 109 genes were definitively categorized into four distinct metabolic pathways: 27 genes were involved in the JH metabolic pathway, 24 in the 20E metabolic pathway, 27 in the chitin metabolic pathway, and 31 in metal transport pathways. A total of 30 genes failed our validation and were temporarily excluded. Furthermore, through RNA sequencing (RNA-seq)-based transcriptome analysis, we observed that under copper (Cu) stress, the expression levels of the majority of genes participating in these metabolic pathways in *P. akamusi* were altered. This finding suggests that copper exposure influences the molting process in *P. akamusi*.

## Simple Summary

Insects rely on molting to grow, a process regulated by hormones like juvenile hormone (JH) and 20-hydroxyecdysone (20E), which control the synthesis and degradation of chitin in their exoskeletons. *Propsilocerus akamusi*, a freshwater midge, is highly tolerant to environmental pollutants, including heavy metals. This study annotates genes related to JH, 20E, chitin metabolism, and metal transport in *P. akamusi*, identifying 139 genes and classifying 109 into specific pathways. Transcriptomic analysis reveals that copper stress alters the expression of these genes, affecting molting processes. Specifically, copper exposure suppresses genes involved in JH synthesis and chitin degradation while inducing genes linked to 20E synthesis and zinc transport. These findings highlight how heavy metals disrupt molting by altering hormone levels and chitin metabolism, potentially causing developmental abnormalities in *P. akamusi*. This research provides insights into the molecular mechanisms of molting and detoxification, offering a foundation for future RNAi-based pest control strategies and environmental toxicology studies.

## 1. Introduction

Insect cuticles form a solid exoskeleton that offers physical protection and effectively minimizes water loss [1]. The rigid exoskeleton is a pivotal factor contributing to the survival of insects. However, because it is a rigid structure, insect growth and development necessitate the periodic shedding or molting of this exoskeleton. This intricate process is tightly regulated through developmental-stage-dependent variations in the concentration profiles of two pivotal insect hormones—the sesquiterpenoid juvenile hormone (JH) and the ecdysteroid 20-hydroxyecdysone (20E) [2]—which are the two primary functional corticosteroid mediators in invertebrate arthropods. Early in each larval instar, JH activity is elevated to maintain the larval features. Conversely, towards the end of each larval instar, the JH titer undergoes a significant decrease, while the 20E titer increases to initiate the molting process [3,4,5,6]. Furthermore, chitin, which serves as the principal component of the exoskeleton, is predominantly located in the epidermal tissues of insects. The cytoplasm that is rich in chitin, associated with the exoskeleton, is termed the cuticle. As the main scaffold component of the stratum corneum, the production and breakdown of chitosan exert a pivotal function in the periodic shedding of the exoskeleton. When chitin is degraded by enzymes such as chitinases, deacetylases, and hexosaminidases and subsequently recycled, the chitinous structure encompassing the epidermis disintegrates, resulting in the shedding of the old exoskeleton. On the other hand, the synthesis of chitin by Chitin Synthase (CHS) initiates the construction of a new exoskeleton, thereby facilitating continuous growth and development in insects.

**Figure 1:**
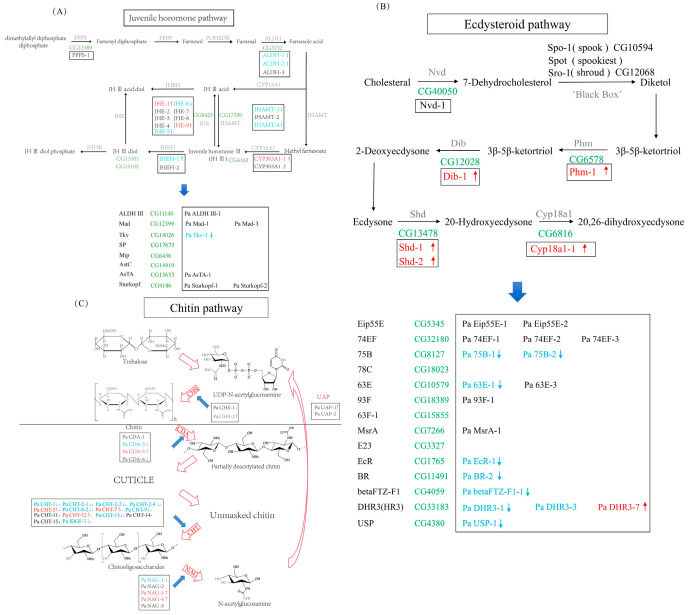
The JH, 20E, and chitin synthesis and degradation pathways in *P. akamusi*, with homologous genes in *P. akamusi* indicated by black boxes and named according to their homologs in *D. melanogaster*. Genes with multiple putative homologs are denoted as -1, -2, -3, and so on. The red arrows beside gene names indicate that the genes are induced in expression under copper (Cu) stress, while the blue arrows indicate that the genes are repressed in expression. (**A**) A JH pathway overview (biosynthesis) in *P. akamusi*. (**B**) A 20E pathway overview (biosynthesis) in *P. akamusi*. (**C**) A chitin pathway overview (biosynthesis) in *P. akamusi*. Genes related to synthesis are located above the black horizontal line, while those related to degradation are below it. The blue-colored font represents genes that are downregulated during Cu stress. The red-colored font represents genes that are upregulated during Cu stress.

JHs are acyclic sesquiterpenoids. Eight different forms of JH have been identified in insects. JH I and JH II are present in the larvae of Lepidoptera, while JH III is found in the majority of insects, with the exception of some Hemiptera species. In higher Diptera, including *Drosophila melanogaster*, in addition to JH III, there is also JH III bisepoxy (JHB3), a JH homolog [7]. The synthesis of JH is linked to the conserved mevalonate pathway (Figure 1A). Firstly, the enzyme farnesyl diphosphate synthase (FPPS) facilitates the condensation of dimethylallyl diphosphate (DMAPP) with two molecules of isopentenyl diphosphate (IPP) in a head-to-tail manner, yielding farnesyl diphosphate (FPP). Following this, FPP undergoes sequential transformations: it is first converted into farnesol by farnesyl diphosphate phosphatase (FPPP), then into farnesal by NADP+-dependent farnesol dehydrogenase (FOHSDR), and ultimately into farnesoic acid (FA) by aldehyde dehydrogenase (ALDH). The transformation of FA into JH can proceed via two distinct pathways. In the first pathway, FA is oxidized by the enzyme methyl farnesoate epoxidase/farnesoate epoxidase (Cyp15A1) to form JH acid (JHA), and JHA is subsequently methylated by juvenile hormone acid methyltransferase (JHAMT) to generate JH [8,9]. Notably, this process is reversible, as JH III can be converted back to JH III acid by the action of juvenile hormone esterases (JHEs). Alternatively, in the second pathway, FA is methylated by farnesoic acid O-methyltransferase (FAMeT) to produce methyl farnesoate (MF). Cyp15A1 then catalyzes the oxidation of MF to yield JH. The metabolic degradation of JH involves three enzymes: JHE, JH esterase hydrolase (JHEH), and juvenile hormone diol kinase (JHDK). Specifically, JH III is first degraded to JH III diol by JHEH. This JH III diol is further degraded to the ultimate product, JH III diol phosphate, by JHDK. Intriguingly, this degradation pathway exhibits a metabolic loop, allowing for the sequential conversion of JH III into JH III diol by JHEH and subsequently into JH III acid diol by JHE. Alternatively, JH III can be first converted into JH III acid by JHE and then into JH III acid diol by JHEH, demonstrating the complexity and flexibility of the JH metabolic pathway [10]. Currently, genes related to JH anabolic metabolism and signal transduction have been characterized in various insect species, including *Tribolium castaneum* (47 genes) [11,12,13], *Danaus plexippus* (44 genes) [14], and *Bombyx mori* (55 genes) [14]. In studies reported thus far, the number of dipteran-related genes is fewer than those of other orders, such as *D. melanogaster* (35 genes) [15,16,17], *Anopheles gambiae* (35 genes) [14], and *Apis mellifera* (36 genes) [18,19].

Ecdysteroids, including ecdyson and 20E, are produced from ingested cholesterol sources via a series of enzymatic reactions [20]. In 1954, Butenandt and Karlson [21] successfully isolated the first ecdysteroid from the pupae of the silkworm, *B. mori*. The production of ecdysone by molting glands occurs in a cyclical fashion, with each secretory peak triggering a molting event. The biosynthetic pathway of ecdysteroids begins with the conversion of the steroidal precursor cholesterol (Figure 1B). Cholesterol undergoes an initial transformation into 7-dehydrocholesterol (7dC) via the catalytic action of the enzyme Neverland (Nvd). Subsequently, 7dC undergoes a complex series of transformations, collectively referred to as the “Black Box,” facilitated by enzymes like Spook (Spo), Spookiest (Spot), and Shroud (Sro), ultimately yielding diketol. The diketol is then converted into 3β,5β-ketodiol by the enzyme Phantom (Phm), which subsequently undergoes further metabolism to produce 3β,5β-ketotriol. The conversion of 3β,5β-ketotriol into ecdysone is catalyzed by the enzyme Disembodied (Dib). Following this, ecdysone undergoes transformation into 20E by the enzyme Shadow (Sad) [22,23]. The degradation of ecdysteroids is predominantly regulated by Cyp18a1, which inactivates 20E through its conversion into 20,26-dihydroxyecdysone [24]. The genes associated with the anabolic metabolism of ecdysteroids are referred to as the Halloween genes, which have been characterized in multiple insect species. The number of Halloween genes varies among species; for instance, *B. mori*, *D. melanogaster*, and *An. gambiae* each contain a complete set of seven Halloween genes [25]. However, in other species such as *Varroa destructor* (three genes), *Bemisia tabaci* (six genes), and *Acyrthosiphon pisum* (five genes), the number of Halloween genes is reduced [26,27,28]. In most insects, Halloween genes are single-copy, except for specific genes in a few insects that contain multiple copies, such as Nvd in *Daphnia magna* [29].

Chitin is a homogenous polymer made up of N-acetylglucosamine. Across various types of insect cuticles, there is minimal variation in its chain length and acetylation. Additionally, extensive research has been conducted on the biosynthetic and degradation pathways of chitin in insects (Figure 1C). The general pathway of chitin synthesis is highly conserved, spanning from fungi to insects. This pathway comprises three distinct subsets of sub-reactions. Firstly, trehalose is hydrolyzed by trehalase (TRE) into two molecules of glucose. Secondly, this glucose undergoes a series of reactions to produce the precursor UDP-N-acetylglucosamine. Finally, CHS removes UDP from the precursor by transfer, resulting in the formation of chitin. In the degradation pathway, chitin undergoes initial degradation into chitooligosaccharides by chitinase (CHT). Following this, β-N-acetylglucosaminidase (NAG) further breaks down the chitooligosaccharides by sequentially removing terminal, non-reducing β-N-acetylglucosamine residues. Chitin undergoes an initial step of degradation where it is converted into chitooligosaccharides by the action of CHT. The coordinated activity of these enzymes is essential for normal insect development. The final product, once activated by UDP-N-acetylglucosamine pyrophosphorylase (UAP), serves as a precursor for the synthesis of new chitin. Finally, chitin deacetylase (CDA) partially deacetylates the chitin, potentially enhancing its resistance to degradation within tissue-specific matrices [30]. CHS is the most crucial enzyme in the synthetic pathway. According to current reports, CHS genes from over 24 other insect species have been identified and cloned, including those from *An. gambiae*, *Ae. aegypti*, *D. melanogaster*, *Manduca sexta*, *T. castaneum*, *Spodoptera frugiperda*, and others [31,32,33,34,35,36,37]. UAP has also been identified in multiple insect species, and most insects possess only a single UAP gene, except for *T. castaneum*, *Locusta migratoria*, and *Locusta decemlineata*. CHT, CDA, and NAG are involved in chitin degradation, with CHT being the rate-limiting enzyme in the chitin degradation pathway, which has been the most extensively studied and has the largest number of gene copies, such as in *An. gambiae* (22 genes), *T. castaneum* (25 genes), *D. melanogaster* (18 genes), *Diaphorina citri* (12 genes), *B. tabaci* (14 genes), and others. NAG, belonging to glycoside hydrolase family 20, has also been identified in many insect species, including *Ae. aegypti*, *B. mori*, *D. melanogaster*, *Pieris rapae*, *Oxya chinensis*, *S. frugiperda*, and so on. Research on CDA is still insufficient. Following the isolation of the first CDA from *Trichoplusia ni* by Guo et al. in 2005 [38], subsequent studies have identified CDA genes in *T. castaneum* (nine genes), *D. melanogaster* (six genes), *An. gambiae* (five genes), and *A. mellifera* (five genes).

Larvae of the Chironomidae family constitute the most prevalent fauna residing in sediments across all freshwater systems, comprising 70 to 80 percent of the total benthic biomass [39]. They are frequently utilized in aquatic ecotoxicology studies due to their roles in food webs, ease of cultivation, close association with surface sediments, and particularly their sensitivity to contaminants and heavy metals [40]. The *Propsilocerus akamusi* is one species of non-biting midge Chironomidae. *P. akamusi* is distributed extensively in freshwater in China and is a dominant species in the chironomid community [41]. Our preliminary research has found that heavy metal exposure can disrupt the developmental process of *P. akamusi*. When exposed to Pb and 20 mg/L of Cu, the molting process of *P. akamusi* larvae occurs prematurely. Currently, there is no research elucidating the pathways of JH, ecdysone, and chitin synthesis and metabolism, as well as heavy metal transport, in *P. akamusi*, and the genes involved in these pathways also lack comprehensive annotation. In our previous study, we assembled and sequenced the genome of *P. akamusi*, obtaining chromosome-level genomic data. A total of 11,110 genes were identified, with 97.6% of conserved arthropod genes present in this genome [42]. Based on previous research, we conducted systematic gene annotation and a functional analysis of the genes involved in these metabolic pathways and excluded some pseudogenes. Additionally, we analyzed the impact of heavy metal stress on the growth and development of *P. akamusi* at the transcriptome level, and identified key responsive genes within each pathway. This study will provide insights into the molecular mechanisms of the molting process in *P. akamusi* and the detoxification processes involving molting-related genes in response to environmental stress. The key responsive genes identified through transcriptome analysis will offer new targets for insect control strategies based on RNAi technology and simultaneously drive the in-depth advancement of insect toxicology research.

## 2. Materials and Methods

### 2.1. Gene Annotation

To identify potential genes in *P. akamusi* that are associated with JH, 20E, and heavy metal transport, we used the relevant proteins from *D. melanogaster* and *An. gambiae* as reference protein sequences (Table S1). Candidate genes in the assembled genome of *P. akamusi* were searched using BLAST+ software v2.16.1 and TBtools-II v2.309 (e-value < 1 × 10^−5^; [43]). The *P. akamusi* genome data utilized for identifying related genes originated from our previous research efforts [44]. Subsequently, we employed the online Pfam 37.0 to authenticate the predicted protein sequences [45]. Among them, the typical JHE (PF00135), CHT (PF00704), ZIP (PF02535), and ZNT (PF01545) proteins are amino acid sequences with unique domains. For the Pfam database accession information of the other genes, we referred to *D. melanogaster*. Specifically for the CHT gene, we employed the SMART v9.0 online tool to perform a validation analysis [46]. We predicted conserved motifs/sequences with the help of the online tools Weblogo (https://weblogo.berkeley.edu/examples.html, accessed on 13 November 2024) and DNAMAN (version 7.212, Lynnon Corp., Vaudreuil-Dorion, QC, Canada) [47]. The prediction of transmembrane (TM) regions in genes was facilitated by the DeepTMHMM 1.0 online tool [48]. The sequences of the genes we annotated were uploaded to Figshare for public access (DOI: https://doi.org/10.6084/m9.figshare.29145170.v1, accessed on 25 May 2025). The genes we annotated in this study referenced the genome assembly data of *P. akamusi* (with the NCBI accession number JAFFZY000000000) [44]. The protein sequences of all the genes we annotated can be found in Table S2, and the accession numbers prefixed with “EVM” (e.g., EVM0001470) are identifiers assigned to genes in the annotation file during the genome annotation process for *P. akamusi*.

### 2.2. Phylogenetic Analysis

The coding DNA sequences (CDSs) of *P. akamusi*, *Homo sapiens*, *Mus musculus*, *D. melanogaster*, *Ae. aegypti*, and *An. gambiae* were aligned altogether using ClustalW within MEGA 11 [49]. All the accession numbers of the proteins used for alignment and analysis are recorded in Table S1. The phylogenetic tree of the CHT gene was constructed using the Poisson model within the Neighbor-Joining (NJ) method, employing amino acid sequences and incorporating 1000 bootstrap replicates. The phylogenetic tree of the JHE gene was constructed using the Jones–Taylor–Thornton (JTT) model within the Neighbor-Joining (NJ) method, employing amino acid sequences and incorporating 1000 bootstrap replicates. The phylogenetic trees for ZIP and ZNT were constructed using the Poisson model within the Maximum Likelihood (ML) method, based on amino acid sequences and incorporating 1000 bootstrap replicates. Figtree and Adobe Illustrator CS6 were utilized for editing and illustrating the trees [50]. The protein accession numbers for all other species mentioned in the article, including *D. melanogaster* and *An. gambiae*, were added to Table S1.

### 2.3. Gene Expression Analysis

Samples of *P. akamusi* were obtained from the Tianjin-located Yongding River, in the People’s Republic of China, and subsequently used for further analysis. Upon arrival, the samples were initially washed three times utilizing purified water before being placed in plastic boxes filled with approximately 1.5 L of chlorine-free municipal water, which had a depth of roughly 3 cm. The samples were aerated for 72 h at a temperature of 22 ± 1 °C with a 16 to 18 h photoperiod. Prior to extracting RNA, the selected experimental samples, which were fourth-instar larvae, were immersed in distilled water for a duration of 24 h. We selected fourth-instar larvae which are highly sensitive to heavy metal stress factors. Copper exposure induces a dose-dependent acceleration of pupation in *P. akamusi*. Our previous research demonstrated that exposure to 10 mg/L, 20 mg/L, and 40 mg/L of CuSO_4_ for 8 days typically results in pupation commencing 2 or 4 days earlier, while exposure to high concentrations of CuSO_4_ leads to high mortality rates and growth disruption. Therefore, the expression levels of molting-related genes were analyzed using expression counts from the experiment conducted previously [44]. In it, *P. akamusi* larvae were exposed to 10 mg/L, 20 mg/L, and 40 mg/L of copper sulfate solution for eight days, during which no feeding was provided. Both the control and exposed groups were handled independently, with each group undergoing three replicates, amounting to a total of 24 samples. We referred to Sun et al.’s study on the expression changes in detoxification genes, heat shock protein (HSP) genes, hemoglobin genes, and chemosensory genes related to metabolic regulation in *P. akamusi* under copper stress for RNA sequencing data [44]. We analyzed the expression levels of genes associated with JH, 20E, chitin metabolism, and heavy metal transport pathways in larvae exposed to a sublethal copper concentration of 20 mg/L. The FPKM values exhibited a wide range, spanning from 0 to over 10,000. Consequently, we categorized gene expression levels according to the following criteria: absent expression (<1), minimal (1–10), low (10–100), moderate (100–1000), elevated (1000–10,000), and very high expression (>10,000) [51,52]. The data on gene expression levels are presented in Table S3. At each time point, we utilized DESeq2 to compare the differentially expressed genes (DEGs) between the samples of the control group and the copper exposure group. We considered genes with a log_2_ Fold Change ≥0.585 or ≤−0.585, and set an adjusted *p*-value threshold (FDR threshold) of 0.1.

## 3. Results

### 3.1. Annotation and Classification of Predicted Genes in P. akamusi

In this study, we annotated and classified the genes related to hormone, chitin, and metal metabolism in the genome of *P. akamusi*, analyzed through sequence similarity to the corresponding genes identified in *An. gambiae* and *D. melanogaster*, and validated these annotations through the presence of certain consensus sequences or motifs. In total, 139 genes related to metabolism were identified. Among them, 109 could be categorized into four metabolic pathways. Specifically, the JH metabolic pathway consisted of 27 genes, 24 genes were in the 20E metabolic pathway, 27 genes in the chitin metabolic pathway, and 31 genes in the metal transport pathway.

### 3.2. Predicted Genes of 20E and JH Synthetic Pathway in P. akamusi

For the biosynthetic pathway of 20E, a total of six transcripts were associated with five enzymes, including Nvd (one transcript), Spo (four transcripts), Sro (three transcripts), Phm (one transcript), Dib (one transcript), and Shd (one transcript). In the 20E degradation pathway, only one transcript was mapped to Cyp18a1 enzymes. For the enzymes regulating 20E metabolism, 17 transcripts were mapped to eleven enzymes, comprising Eip55E (2 transcripts), 74EF (3 transcripts), 75B (2 transcripts), 63E (2 transcripts), 93F (1 transcript), MsrA (1 transcript), EcR (1 transcripts), BR (1 transcript), betaFTZ-F1 (1 transcript), DHR3 (2 transcript), and USP (1 transcript). The Halloween genes participate in the biosynthesis of 20E and belong to the P450 gene family, exhibiting evolutionary conservation across almost all insect species. To date, nearly all known insect genomes possess a clear single ortholog of these genes, with the exception of *A. pisum*, which contains a duplicated gene for Shd. Most of the genes, including Eip55E, 74EF, 75B, 63E, 93F, MsrA, EcR, BR, betaFTZ-F1, DHR3, and USP, affect the anabolic process of 20E by participating in the ecdysone signaling pathway. For instance, the protein encoded by EcR can form a heterodimer with USP, functioning as a receptor for 20E.

Within the biosynthetic pathway of JH, a total of 10 transcripts were mapped to five enzymes, including FPP (1 transcript), ALDH (4 transcripts), JHAMT (3 transcripts), and CYP15A1 (2 transcripts). In the degradation pathway of JH, a total of 11 transcripts were associated with three enzymes, specifically JHE (9 transcripts) and JHEH (2 transcripts). In the enzymes regulating JH metabolism, six transcripts were mapped to four enzymes comprising Mad (two transcripts), TKV (one transcript), AsTA (one transcript), and Sturkopf (two transcripts). In insects, FPP can be utilized for the synthesis of ubiquinone, polyols, protein isoprenoids, and other substances; it serves as a precursor for JH. ALDH catalyzes the oxidation of aldehydes into corresponding carboxylic acids and is a crucial enzyme for the detoxification of various endogenous, exogenous, and lipid peroxidation products containing highly reactive aldehydes in insects. For instance, ALDH is involved in acetaldehyde detoxification in *D. melanogaster* [53] and pesticide detoxification in *Ae. aegypti* [54]. JHAMT is the rate-limiting enzyme for JH synthesis. Numerous studies have demonstrated that gene knockout or the knockdown (silencing) of JHAMT expression in insects leads to growth disorders, reduced reproductive quality, diapause, and other phenotypic effects [13,55]. Compared to JHAMT, there are relatively fewer studies on CYP15A1, and the silencing effect of RNAi on it is also less effective. The degradation of JH primarily occurs through three enzymes: JHE, JH esterase hydroxylase (JHEH), and JH dihydroxyketone reductase (JHDK). The methyl ester of JH is hydrolyzed by JHE, which is the most crucial and extensively studied enzyme among the three involved in JH degradation, primarily functioning in the hemolymph of insects to degrade JH. Promoting or inhibiting JHE activity at the target stage can affect JH levels and lead to abnormal development and growth. Xu et al. [56] used RNAi to reduce JHE expression in adult *T. castaneum*, resulting in dwarfism and reduced feathering rates [57]. Additionally, JHE has been a target in the development of biological insecticides due to its selectivity across insect orders and even families, which is unparalleled by traditional insecticides. The epoxide of JH is cleaved by JHEH, which is located within tissues and membrane-bound. Compared to JHE, there are relatively fewer studies on JHEH in insects. The Tkv-Mad pathway in the TGFβ signaling cascade influences JH synthesis by affecting the transcription of JHAMT. AsTA is a neuropeptide that inhibits JH synthesis, while Sturkopf interacts with JHEH to affect the degradation of JH.

#### Juvenile Hormone Esterase

Insect chitinases play a pivotal role as enzymes responsible for the breakdown of chitin within the exoskeleton during the ecdysis phase [58]. They also exhibit carboxylesterase activity, capable of hydrolyzing reproductive hormone JH, thereby partially regulating its titer. JHEs catalyze the reversible conversion of JH to JH acid [59,60,61]. JHE is the most critical and widely studied of the three enzymes involved in JH degradation, primarily responsible for degrading JH in the hemolymph of insects.

Up to now, genes that encode JHEs or putative JHEs have been identified in diverse insect species. These enzymes possess characteristic motifs, including RF, DQ, E, and GxxHxxD/E, where “x” represents any amino acid [18,62,63]. Additionally, the presence of the GxSxG motif, specifically in the form of GQSAG, is a vital component within JHEs.

In *P. akamusi*, nine JHEs were identified and annotated, and Pa JHE-5 and Pa JHE-9 were localized on chromosome 1, Pa JHE-8 was localized on chromosome 3, Pa JHE-3 was localized on chromosome 4, and the others were all localized on chromosome 1. The results of ClustalX analysis show that the protein sequence of Dm JHE shows 25.81~44.09% identity to Pa JHE.

**Figure 2:**
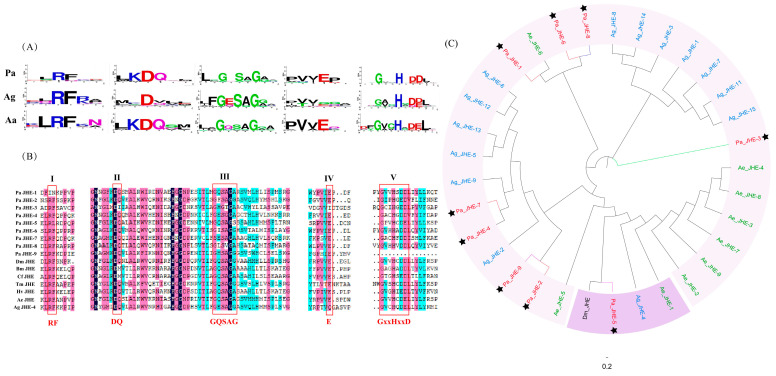
The JHE genes in *P. akamusi*, *Ae. aegypt*, *An. gambiae*, and *D. melanogaster*. (**A**) The WebLogos analysis of the five conserved regions for the JHE proteins of *P. akamusi*, *Ae. aegypt*, *An. gambiae*, and *D. melanogaster.* The conservation of the five conserved regions of JHE in *Ae. aegypt* and *P. akamusi* is higher than that in *An. gambiae*. The red stars mark the highly conserved residues. (**B**) The conserved (classical) core catalytic sequences of JHE in the *P. akamusi* genome. In *P. akamusi*, all the putative genes for JHE contain multiple classical core catalytic sequences typical of JHE. A darker background color indicates higher conservation of the amino acid at that position. (**C**) A phylogenetic tree of annotated JHE proteins among *P. akamusi*, *Ae. aegypt*, *An. gambiae*, and *D. melanogaster*. Pa JHE-5 clusters with physiologically active JHEs from *Ae. aegypt*, *An. gambiae*, and *D. melanogaster*, suggesting that it may be the physiologically active JHE in *P. akamusi*. The Pa JHE genes are marked with black stars. The hues of the branch lines indicate the chromosome location of the JHE genes: specifically, chromosome 1 is designated in crimson, chromosome 2 in verdant green, chromosome 3 in azure blue, and chromosome 4 in a soft lilac hue.

We examined the Pa JHE 1–9 genes to ascertain if they maintained five crucial motifs (Figure 2B). Pa JHE contains RF, DQ, GQSAG, E, and GxxHxxD/E motifs. Pa JHE-2, Pa JHE-4, Pa JHE-5, Pa JHE-6, Pa JHE-7, and Pa JHE-8 have all of these motifs, while Pa JHE-1 lacks the RF motif, Pa JHE-3 lacks the E motif, and Pa JHE-9 lacks the GxxHxxD/E motif. The DQ motif was substituted by DI in Pa JHE-3. The first glycine residue of the GxSxG motif present in Pa JHE-7 was changed to Serine, and the Serine residue of the GxSxG motif present in Pa JHE-3 was changed to glycine. Interestingly, Pa JHE-1 and Pa JHE-5 harbor the GQSAG sequence, which is the prototypical motif of JH-selective esterases. In addition, the Aspartic acid residue of the GxxHxxD motif present in Pa JHE-5 was changed to Glutamic acid. We compare these five conserved motifs of *P. akamusi*, *An. gambiae*, and *Ae. aegypti*. According to the WebLogos analysis of these motifs, we find that the RF motif is highly conserved in these three species, and that the DQ and E motifs of *P. akamusi* and *Ae. aegypti* are more conserved than in *An. gambiae* (Figure 2A). The Aspartic acid residue of the GxxHxxD motif present in Ae JHE and partial Ag JHE was changed to Glutamic acid. In addition, the GxSxG motifs of these three species possess greater variability in the second amino acid.

The analysis of genomic sequences reveals that dipteran genomes likely possess a coding capacity for multiple JHE genes. Although multiple JHE genes may exist in their genomes, functional assessments of the proteins they encode indicate that each dipteran genome probably expresses only one physiologically functional JHE. In the case of *D. melanogaster*, for instance, its genome encodes four esterases exhibiting JHE-like properties; however, only one of these enzymes demonstrates physiological functionality as a JHE [64]. Analogously, the genome of *Ae. aegypti* harbors ten esterases that possess JHE-like attributes, yet only one among them manifests as a physiologically active JHE [65].

To compare the JHEs of *P. akamusi* with those of other Diptera species, we aligned 33 JHE protein sequences from *D. melanogaster*, *P. akamusi*, *Ae. aegypti*, and *An. gambiae* using ClustalX (Figure 2C). Phylogenetic analysis revealed that Pa JHE-5 clusters with physiologically active JHEs from *D. melanogaster* and *Ae. aegypti*. Notably, Pa JHE-5 exhibits the highest sequence identity (44.09%) to *D. melanogaster* JHEs among all *P. akamusi* JHEs, suggesting that it may retain ancestral core catalytic functions critical for conserved physiological processes such as development or hormone regulation in *P. akamusi*. Thus, Pa JHE-5 likely represents the physiologically active JHE in this species. On the phylogenetic tree, *D. melanogaster* JHEs form a distinct clade, indicating that their gene family has undergone limited gene duplication events and evolved under strong functional conservation. In contrast, JHEs from *Ae. aegypti* and *An. gambiae* diverge into two independent subclades, potentially reflecting adaptations to distinct environmental stresses (e.g., blood-feeding, pathogen transmission) that necessitate stage-specific JHE functions. Remarkably, *P. akamusi* JHEs are interspersed with those of the other three species. This pattern suggests that chironomid JHEs have evolved under complex selective pressure combinations—such as aquatic–terrestrial habitat shifts or dietary specializations—driving functional divergence. While some Pa JHEs (e.g., Pa JHE-5) retained their ancestral roles, others underwent adaptive evolution to meet novel ecological demands, highlighting the dynamic interplay between functional conservation and innovation in insect hormone regulatory systems.

### 3.3. Predicted Genes of Chitin Synthetic Pathway in P. akamusi

Within the biosynthetic route of chitin, 23 transcripts were assigned to five enzymatic proteins, including CDA (4 transcripts), CHT (14 transcripts), and NAG (5 transcripts). In the chitin degradation pathway, four transcripts were mapped to two enzymes, including UAP (two transcripts) and CHS (two transcripts). The degradation of chitin plays a pivotal role in the process of shedding insect exoskeletons. Chitinases are capable of decomposing chitin into chitooligosaccharides, which can subsequently be utilized for the synthesis of new chitin molecules [30,66]. CHS and UAP are involved in chitin synthesis. CHS belongs to the metalloprotein family of extracellular chitin-modifying enzymes. In insects, CHS genes are classified into two categories: CHS1 and CHS2. CHS1 is primarily responsible for synthesizing chitin in epidermal and tracheal cells, while CHS2 is produced by midgut epithelial cells to form the peritrophic matrix (PM). UAP catalyzes the production of an important substrate for chitin formation. CHT, NAG, and CDA are involved in chitin degradation. CHT is an endochitinase that hydrolyzes chitin polymers into smaller oligosaccharides. NAG belongs to the carbohydrate-active enzyme glycoside hydrolase family 20 and is an exochitinase that can further cleave these oligosaccharides into N-acetylglucosamine monomers. They degrade chitin through synergistic effects. CDA catalyzes the deacetylation of various carbohydrate substrates and has specific functions in the morphogenesis of chitin-containing body parts and the survival of insects.

#### Chitinase

The number of chitinase genes and their expression display significant variations among different species. Within the genetic blueprints of insects and arthropods of the crustacean class, the count of chitinase genes ranges from 3 to 24 [67,68,69,70,71]. Utilizing phylogenetic analysis and the conservation of protein domains, chitinases are categorized into ten distinct groups (I–X) [72]. The number of glycoside hydrolase 18 (GH18) catalytic domains and chitin-binding domains (CBDs) varies among these 10 groups (I–X) of chitinases. CHTs (chitinases) exhibit unique expression patterns specific to tissues and/or developmental stages. Chitinases from Groups I, II, III, V, VI, VII, and VIII are expressed almost throughout all developmental stages, including embryos, larvae, pupae, and adults. In contrast, chitinases belonging to Group IV are only expressed during the larval and adult stages, but not during the prepupal, pupal, or embryonic stages. All the genes in Group IV are expressed in the larval intestinal tissue but not in the carcass (the whole body excluding the intestine and head). Chitinase-like proteins from Group V have been shown to regulate cell proliferation and remodeling in both insect and mammalian cells. Functions vary greatly among the groups. For example, RNAi of CHT genes characterized in *T. castaneum* has demonstrated that enzymes from Groups I and II are involved in digesting epidermal chitin, while CHTs from Group III regulate wing expansion and abdominal contraction. Furthermore, some members of Group V are essential for adult emergence. Different chitinases are expressed at different developmental stages. Insect chitinases harbor four conserved regions within their GH18 domains. Specifically, Conserved Region I (CR_I) is represented by the sequence KxxxxxGGW, while Conserved Regions II (CR_II), III (CR_III), and IV (CR_ IV) correspond to the sequences FDGxDLDWEYP, MxYDxxG, and GxxxWxxDxDD, respectively [73,74].

**Figure 3:**
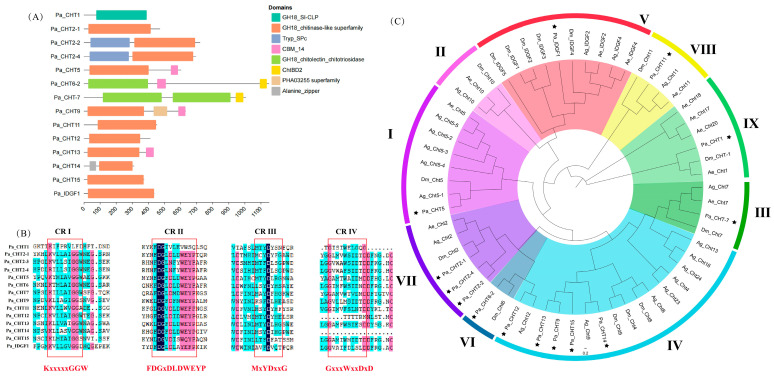
The CHT genes in *P. akamusi*, *Ae. aegypt*, *An. gambiae*, and *D. melanogaste*. (**A**) The domain architecture of the identified *P. akamusi* chitinase-like proteins. Each protein possesses the CH18 domain. (**B**) The conserved (classical) core catalytic sequences of CHT in the *P. akamusi* genome. Each protein contains 1 to 4 core catalytic sequences. A darker background color indicates higher conservation of the amino acid at that position. (**C**) A phylogenetic tree of annotated CHT genes from *P. akamusi*, *Ae. aegypt*, *An. gambiae*, and *D. melanogaste*. The CHT genes of *P. akamusi* can be clearly classified into different groups, and there are no genes belonging to Group II and Group X in *P. akamusi*. The black stars indicate the Pa CHT genes.

Here we have identified 14 CHT genes in *P. akamusi*. These 14 CHTs all possess the CH18 domain (Figure 3A). Subsequently, we constructed a phylogenetic tree for *P. akamusi*, *D. melanogaster*, *An. gambiae*, and *Ae. aegypti*. According to the phylogenetic tree, these 14 CHTs were classified into nine distinct groups (Figure 3C). Analogous to the majority of insects, only one chitinase belonging to Group I (Pa CHT-5) was detected in *P. akamusi*. Notably, while the gene duplication of Group I chitinases has been observed in *An. gambiae*, such duplication is absent in both *P. akamusi* and *Ae. aegypt*. Despite the presence of Group II chitinases in species such as *D. melanogaster*, *An. gambiae*, and *Ae. aegypti*, no Group II chitinases were identified in *P. akamusi*. Furthermore, one chitinase each from Groups III, V, VI, VIII, and IX was identified in *P. akamusi*. In holometabolous insects, Group IV chitinases represent the largest and most varied category [66]. Five Group IV chitinases were identified in *P. akamusi*, suggesting the presence of gene duplication within this group in this species. Additionally, we did not identify any Group X chitinase genes in *P. akamusi*, Similarly, this absence was also observed in the genomes of *D. melanogaster*, *An. gambiae*, and *Ae. Aegypt*. Group X genes seem to have been lost in the Diptera lineage [72].

The comparison results indicated that the CR_I and CR_II of genes I–VIII exhibited a high degree of conservation (Figure 3B). The chitinases of *P. akamusi* had greater variability in the last amino acid of CR_I, and the conserved region of CR_2 was (F/L)DGx(D/N)xx(W/Y)(E/Q)(Y/F)P. Compared to the FDGxDLDWEYP motif, *P. akamusi* have greater variability on the sixth and seventh amino acids. The conservation of CR_III and CR_IV motifs in insects was found to be lower compared to CR_I and CR_II. Specifically, in *P. akamusi*, the first amino acid M in CR_III of Pa CHT6-1, Pa CHT-15, and Pa CHT-9 was substituted with L, L, and K, respectively, while the amino acids in other positions remained well-preserved. Notably, the CR_IV motif was absent in Pa CHT-12 and Pa CHT-14, a phenomenon that was also observed in *D. melanogaster* and *An. gambiae*.

### 3.4. Predicted Genes of Metal Transporter Pathway in P. akamusi

The ontogeny and maturation of insects are closely linked to metal ions, as numerous insect proteins require metals to fulfill their functions. However, an excess of metals can be detrimental to cells. To maintain stable metal concentrations within cells, insects possess specific transporters for different metals, which work in coordination and cooperation with each other. In this study, we identify 31 genes in the metal transport pathway in *P. akamusi*. Their transcripts were mapped to twelve enzymes, including ZIP (seven transcripts), ZNT (six transcripts), Tsf1 (four transcripts), Ccs (three transcripts), Nemy (two transcripts), Ferritin (two transcripts), Mvl (one transcript), Mfrn (one transcript), Fh (one transcript), Cox17 (one transcript), Cox11 (one transcript), ATP7 (one transcript) and MTF-1 (one transcript).

#### 3.4.1. Metal Transporter 1 (DMT1)

**Figure 4:**
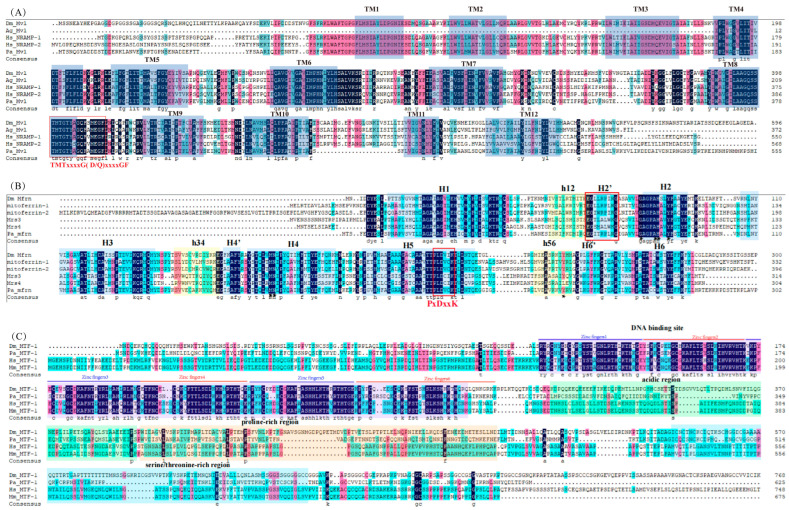
The sequence analysis of *P. akamusi* Mvl, Mfrn, and MTF-1. The shaded black, pink, and blue areas show complete sequence identity and similarity. (**A**) The amino acid sequence alignment of *P. akamusi*, *H. sapiens*, and *D. melanogaster* Mvl. Pa Mvl possesses the transmembrane domain and classic consensus sequence that are unique to Mvl proteins. The blue shading indicates the 12 transmembrane (TM) domains of Pa Mvl, while the red boxes highlight the classic consensus sequence of PaMvl: TMTx(4)GD/Q(4)GF. (**B**) An alignment was conducted for the amino acid sequences of mitoferrin from *P. akamusi* (Pa Mfrn), *D. melanogaster* (Dm Mfrn and CG4963), zebrafish (Mfrn1 and Mfrn2), and yeast Mrs3p and Mrs4p. Pa Mfrn has the substrate-binding region, potential active sites, and transmembrane domain that are characteristic of Mfrn proteins. The sequences are annotated, with arrowheads indicating substrate-binding regions and black stars indicating potential interaction sites. Additionally, markers are positioned above the sequences to highlight the transmembrane domains H1-H6 and matrix-facing helices h12, h34, h56. (**C**) The amino acid sequence alignment of *P. akamusi*, *D. melanogaster*, *H. sapiens*, and *M. musculus* MTF-1. Pa MTF-1 features the nuclear export signal (NES), nuclear localization signal, and putative domains that are specific to Mvl proteins. Dashes are incorporated to bridge the gaps for achieving optimal sequence alignment. In addition, nuclear export signal (NES) and nuclear localization signal (NLS), which fit the consensus motif KQREVKR, were also located. The putative domains are shown in the blue shaded area.

Iron (Fe) can be absorbed in its ferrous (Fe^2+^) or ferric (Fe^3+^) state. Primarily, Fe^2+^ is taken up by the divalent metal transporter 1 (DMT1) [75]. DMT1 transports a wide variety of substances, such as zinc (Zn), copper (Cu), magnesium (Mg), and toxic metals like lead (Pb) and cadmium (Cd) [76]. In *P. akamusi*, one Mvl ortholog has been identified and annotated. ClustalX analysis reveals that Pa Mvl-1 shares 61.66% identity with Dm Mvl and 62.2% identity with Ag Mvl. Furthermore, transmembrane helix prediction by Pfam confirms that Pa Mvl-1, like other Mvl family members, possesses 12 TM domains. The alignment results from ClustalX show that the Mvl of *P. akamusi* contains the following classic consensus sequence: TMTx(4)GD/Q(4)GF (Figure 4A). In line with other DMT1 orthologs, the classic consensus sequence regions exhibit strong conservation between *D. melanogaster* and *P. akamusi*. Additionally, among the investigated species of *P. akamusi* and *D. melanogaster*, the GD motif is more prevalent than the GQ motif within the classic consensus sequence.

#### 3.4.2. Mitoferrin (Mfrn)

Upon the entry of Fe^2+^ into the cytoplasm, it can be transported into mitochondria by mitoferrin-1. In *P. akamusi*, we identified a single ortholog of Mfrn. When aligning the amino acid sequence of Pa Mfrn with yeast Mrs3/4p and zebrafish Mfrn1 and Mfrn2, we noted a greater level of sequence preservation amongst the proteins of metazoans, ranging from 10.95% to 16.01%. Specifically, Pa Mfrn exhibited 54.96%, 52.16%, and 52.00% sequence similarity to Dm Mfrn-1, Mfrn1, and Mfrn2, respectively. In contrast, the sequence identity between Pa Mfrn and the yeast proteins (Mrs3p and Mrs4p) was 41.05% and 38.95%, with a sequence similarity of 41% for both yeast proteins (Figure 4B). Robinson and Kunji have suggested a proposed binding site for substrates, along with three interaction points, within mitochondrial carrier proteins [77,78]. The binding site for the putative substrate, along with contact sites I and II, are consistent across Pa Mfrn, Dm Mfrn, Mrs3/4p, and Mfrn1/2. Meanwhile, contact site III exhibits identity between the two yeast mitochondrial carriers. Additionally, the Pfam results revealed that Pa Mfrn-1 possesses a tripartite structure. According to the alignment results, Pa Mfrn-1 contains six transmembrane α-helices, three short α-helices, and the following conserved motifs: PX(D/E)xx(R/K) and (D/E)Gxxxx(W/Y/F)(R/K)G. However, in Pa Mfrn-1, the first amino acid in the (D/E)Gxxxx(W/Y/F)(R/K)G motif is E, resulting in the motif EGITRPLRG, instead of the typical (D/E)Gxxxx(W/Y/F)(R/K)G. Similarly, the third amino acid in the PX(D/E)xx(R/K) motif is D and the sixth amino acid is K, leading to the motif PLDVCK rather than the standard Px(D/E)xx(R/K).

#### 3.4.3. Frataxin (FXN) and No Extended Memory (Nemy)

Fe^2+^ entering mitochondria can further bind to frataxin (FXN), an essential Fe-homeostasis-related protein located in mitochondria. We identified a single frataxin gene in the genome of *P. akamusi*. Pa Fh shares 46.01% identity with the *D. melanogaster* frataxin putative ortholog and 44.83% with *An. gambiae*. The alignment outcomes indicate a significant similarity in their amino acid sequences.

Iron in its Fe^3+^ form needs to be reduced to Fe^2+^ by ferroreductases such as duodenal cytochrome b (Dcytb) before it can be absorbed into cells. Dcytb possesses ferric reductase activity, converting dietary inorganic iron from its Fe^3+^ state to the Fe^2+^ state. In *D. melanogaster*, two candidate genes, no extended memory (Nemy) and CG1275, have been identified for this function.

Based on sequence homology with the *D. melanogaster* genes, we annotated the Nemy and CG1275 genes from the genome of *P. akamusi*. A Clustal Omega-mediated alignment between the *P. akamusi* and *D. melanogaster* Nemy proteins showed a significant similarity in their polypeptide chain compositions, with an identity of 54.33%. Similarly, Pa-CG1275 shares 55.51% identity with Dm CG1275. Proteins belonging to the Dcytb family typically have six transmembrane helices. The Pfam domain analysis of *P. akamusi* genes shows that they possess six TM domains, consistent with the characteristics of the Dcytb family.

Both Nemy and CG1275 serve as ferric reductases, featuring a core domain of cytb561 (TM2–TM5) composed of four consecutive helices [79,80]. This domain is stabilized through coordination by four conserved histidine residues that are universally present. However, unlike Nemy proteins, CG1275 proteins contain an “EDxxLL” motif located between TM6 and the C-terminus [81] (Figure S1).

#### 3.4.4. Ferritin

Upon being imported into mitochondria, one of the most crucial mechanisms for iron storage is ferritin. This ubiquitously expressed protein serves dual functions: detoxification and storage. In *P. akamusi*, we identified two paralogs of ferritin. The Pa Ferritin-1 protein sequence exhibited 49%, 31.46%, and 44.67% identity with Dm Ferritin-1, Dm Ferritin-3, and Ag Ferritin-1, respectively. Similarly, the Pa Ferritin-2 protein sequence showed 32.11% and 28.7% identity with Dm Ferritin-2 and Ag Ferritin-2, respectively (Figure S2). Sequence alignment reveals that the amino acid sequences of Pa Ferritin are semi-conserved or conserved compared to those of known ferritins, suggesting that insect ferritin might be less stable or stabilized by additional factors.

Furthermore, Fe^3+^ can form a complex with transferrin (TF) in the plasma for transport, known as the transferrin receptor (TfR) cycle. The Fe^3+^—transferrin complex binds to transferrin receptor 1 (TfR1) and is internalized into endocytic vesicles [82]. Inside the vesicles, Fe^3+^ is reduced to Fe^2+^ by a ferroreductase [83] and then further enters the cytoplasm through DMT1 on the endosomal membrane [83,84].

#### 3.4.5. MTF-1

The MTF-1/MT system serves as the primary regulatory mechanism for maintaining intracellular metal homeostasis and responding to heavy metal loads. Under normal physiological conditions, MT expression remains at a baseline level but can be significantly induced in the presence of heavy metals. As the cellular concentration of heavy metals like Cd, Pb, and Hg rises, they substitute for the Zn cofactor in metallothionein, resulting in the liberation of free Zn ions. Subsequently, these Zn ions attach to MTF-1, allowing it to engage with metal-responsive motifs (MRMs) positioned within the regulatory sequences of target genes, including promoters or enhancers, which in turn triggers the expression of downstream genes.

Based on sequence similarity with *D. melanogaster*, we annotated the MTF-1 genes of *P. akamusi*. Clustal Omega (https://www.ebi.ac.uk/jdispatcher/msa/clustalo, accessed on 8 July 2024) analysis shows an identity of 44.01, 35.31, and 37.70% with that of *D. melanogaster*, *H. sapiens* and *M. musculus*, respectively. There is a high degree of homology in their amino acid sequences. MTF-1 is a protein characterized by six C2H2-type zinc fingers, which are crucial for its role in the heavy metal response [85]. The alignment reveals that Pa MTF-1 possesses a distinctive N-terminal segment, succeeded by a DNA-binding domain that comprises six C_2_H_2_-type zinc fingers (Figure 4C). The C-terminal portion of the protein harbors a transactivation domain, comprising three presumably separate activation subdomains: an acidic segment, a proline-dense domain, and a serine/threonine-abundant region. Figure 4C highlights the conservation of the functional domains, especially the DNA-binding domain of *P. akamusi* MTF-1, with that of other species analyzed. The zinc finger DNA-binding domain, involved in specific DNA recognition, is found to be the most conserved part, followed by the transactivation domain.

#### 3.4.6. Cox17

Copper, as a transitional element, is essential for typical growth and maturation processes [86]. It serves as an essential cofactor for a wide range of enzymes. The preservation of copper homeostasis is dependent on the functioning of copper transporters that facilitate the intake and expulsion of the metal from cells, as well as copper chaperones that direct it to precise destinations within the cell [87]. In the blood, ceruloplasmin transports Cu2+ to the reductase situated on the cell membrane, where it is reduced to Cu+. This Cu+ is then taken into the cell by CTR1 (copper transporter 1), a membrane-associated transporter. CTR1 serves as the primary transporter for copper entry into cells [88,89]. Upon entering the cell, copper is transported by three key copper chaperones—Atox1, Ccs, and Cox17—which facilitate its delivery to copper-dependent enzymes [90,91].

Here we annotated three Ccs genes and one Cox17 gene from the genome of *P. akamusi* based on sequence homology with *D. melanogaster*. However, we did not annotate the homologous proteins CTR1 and Atox1 in *P. akamusi*. The similarities between Dm Cox-17 and Pa Cox17, Ag Cox17-1 and Pa Cox17, and Ag Cox17-2 and Pa Cox17 were as high as 60%, 59.46%, and 61.97%, respectively. Similarly, the similarities between Dm Ccs and Pa Ccs-1, Dm Ccs and Pa Ccs-2, and Ag Ccs and Pa Ccs-1 were also very high, at 46.48%, 41.89%, and 44.04%, respectively. The alignment results revealed that all the crucial residues identified by Punter and Glerum (2003) [92], specifically Cys-23, Cys-24, Cys-26, Asp-34, Cys-47, and Cys-57 (based on *S. cerevisiae* numbering), were conserved in the *P. akamusi* sequences, with the exception of Arg-33, which was substituted by Lys in *P. akamusi*. Cox17 contains a concise shared motif featuring the sequence KxCCxC, which is a putative copper-binding site. This common domain was also present in the *P. akamusi* sequences (Figure S3).

#### 3.4.7. ATP7

ATP7 proteins are responsible for transporting copper into the trans-Golgi network (TGN) and for its excretion from cells. The Clustal Omega-mediated alignment between Dm ATP7 and Pa ATP7 proteins revealed a strong homology in their amino acid sequences (ID: 60.26). According to the Pfam results, Pa ATP7 contains eight transmembrane domains, which is consistent with other ATP7 proteins. The N-terminal portion of ATP7 contains six repetitive sequences (MBRs), each carrying the metal-binding motif GMT/HcxxCxxxIE [93].

We utilized Clustal to examine the amino acid sequences of Dm ATP7 and Pa ATP7, revealing that, in addition to the sequence motifs common to all P-type ATPases, Pa ATP7 exhibits unique structural attributes associated with its specific role in copper transport. Specifically, Pa ATP7 contains five membrane-spanning regions (MBRs), and the metal-binding motifs are characterized by the sequence GMxCxxCxxxIE. However, we found that two of the MBRs in *P. akamusi* were GMKCxxCxxxIE instead of GMTCxxCxxxIE (Figure S4).

#### 3.4.8. ZIP Transporter

Zinc is indispensable for the growth of all life forms. Cells primarily facilitate the transport of zinc through ZIP and ZNT transporters. ZIP transporters are responsible for facilitating zinc translocation across membrane barriers into the cytosolic compartment, enabling the entry of zinc from the extracellular milieu as well as facilitating its efflux from intracellular organelles. According to the resemblance in amino acid sequences, the ZIP family can be categorized into four subfamilies: ZIPI, ZIPII, gufA, and LIV-1 [94]. A shared characteristic among these four subfamilies is the existence of an extended, diverse region within the cytoplasmic loop positioned between transmembrane domains III and IV. This loop exhibits variability and contains a histidine-rich repeat, defined by the pattern (Hx)n, where H stands for histidine, x signifies any amino acid, and n has a range from 3 to 6 [95,96,97]. While most ZIP members possess eight predicted TM domains, a portion of the LIV-1 subfamily boasts only six TMDs [98]. The ZIPI subfamily exhibits conserved sequences, namely Lx(2)Hx(4)GxAxG and HKx(4)F, within TM III and IV. Notably, unlike the other subfamilies, members of the LIV-1 family, exclusively found in eukaryotic cells, share the HExPHExGD motifs within TM V [98].

**Figure 5:**
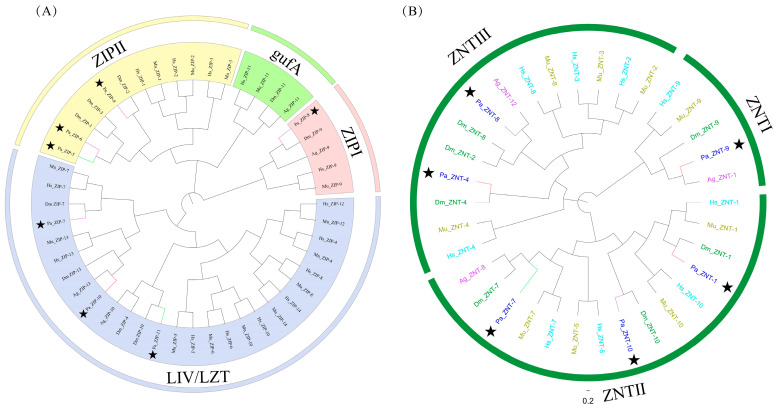
The ZIP and ZNT genes in *P. akamusi*, *H*. *sapiens*, *D. melanogaster*, *M. musculus*, and *An. gambiae*. (**A**) A phylogenetic analysis of the annotated ZIP genes from *P. akamusi*, *D. melanogaster*, *An. gambiae*, *H. sapiens*, and *M. musculus*. Among the seven ZIP genes in *P. akamusi*, they are classified into three different ZIP subfamilies; however, no genes belonging to the gufA family are identified in *P. akamusi*. (**B**) A phylogenetic analysis of the annotated ZNT genes from *P. akamusi*, *D. melanogaster*, *An. gambiae*, *H. sapiens*, and *M. musculus*. The six ZIP genes in *P. akamusi* can be well classified into three different ZNT subfamilies. The black stars indicate the genes of *P. akamusi*. The hues of the branch lines signify the chromosome where the ZIP and ZNT genes reside. Specifically, chr 1 is denoted by red, chr 2 by green, and chr 4 by lilac.

Here we annotated the ZIP genes in the genome of *P. akamusi* based on their sequence homology to the confirmed ZIP genes in *D. melanogaster*. According to the results obtained from Pfam, we analyzed 11 ZIP genes of *P. akamusi* and ultimately identified 7 Pa ZIP genes: Pa ZIP-7, Pa ZIP-10, and Pa ZIP-11 possess six TM domains, while Pa ZIP-5, Pa ZIP-6, and Pa ZIP-9 have eight TM domains. All genes, except for Pa ZIP8, possess an (HX)n repeat rich in histidine within the variable segment of the cytoplasmic loop positioned between transmembrane domains III and IV. The alignment performed by Clustal Omega between Dm ZIP and Pa ZIP indicated a high degree of homology in their amino acid sequences. To classify the ZIP gene in *P. akamusi*, we constructed a phylogenetic tree using ZIP proteins from *P. akamusi*, *Homo sapiens*, *D. melanogaster*, *M. musculus*, and *An. gambiae* (Figure 5A). Phylogenetic analysis indicates that the Pa ZIP family is divided into subfamilies ZIPI, ZIPII, and LIVl/LZT. Pa ZIP-7, Pa ZIP-10, and Pa ZIP-11 blong to the LIV-1 family; Pa ZIP-5, Pa ZIP-6, and Pa ZIP-9 belong to the ZIPII family; and Pa ZIP-8 belongs to the ZIPI family. However, we did not detect any members of the gufA family within Pa ZIP.

The alignment results reveal that Pa ZIP-7, Pa ZIP-10, and Pa ZIP-11 possess the classic consensus sequence of the LIV-1 family—HExPHExGD—in TMs IV and V (Figure S5). Pa ZIP-5, Pa ZIP-6, and Pa ZIP-9 and Dm ZIP-1, Dm ZIP-2, and Dm ZIP-3 have high degrees of similarity, and these proteins have conserved sequences Lx(2)Hx(4)GxAxG and HKx(4)F in TMs III and IV. It is interesting that the conserved sequences of Pa ZIP-8, Dm ZIP-9, Hs ZIP-9, and Ms ZIP-9 are LVVHAAADGxALG and HKAPAAF and not Lx(2)Hx(4)GxAxG and HKx(4)F in TMs III and IV (Figure S6). The sequence results of these genes align with the characteristics of each subfamily.

#### 3.4.9. ZNT Transporter

ZNT transporters facilitate the movement and storage of zinc away from the cytosol, directing it towards intracellular organelles and vesicles, in contrast to the function of ZIP transporters [99]. So far, ten CDF proteins, which are named ZNT proteins and originate from either humans or mice, have been identified or had their molecular characteristics determined [100,101,102]. Based on their sequence similarities, ZNT transporters are categorized into three subgroups: ZNT subfamily I, ZNT subfamily II, and ZNT subfamily III [94]. A majority of ZNT transporters possess six transmembrane domains (TMDs) [103]. Unlike ZIP proteins, ZNT proteins are distinguished by an elongated histidine-abundant loop located between TMDs IV and V. This loop is described by the formula (Hx)n, with n varying from 3 to 6, potentially serving as a metal-binding site. The histidine-rich loop is thought to be pivotal as a metal-binding site and performs vital functions. The highly amphipathic natures of TMDs I, II, and V are well-preserved. TMDs I, II, and V exhibit the highest degrees of conservation among CDF proteins.

**Figure 6:**
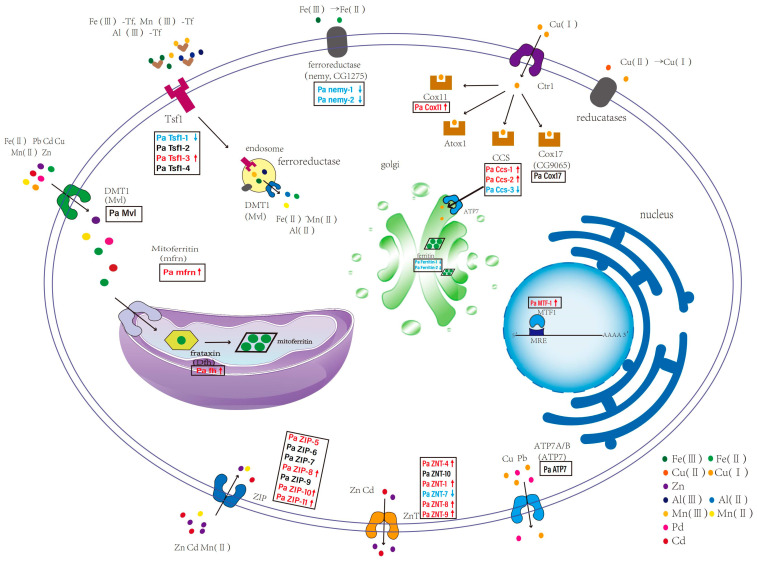
The known pathways of transport and homeostasis regulation of different metal ions in Drosophila and Chironomidae cells. The Arrows present the direction of the metal transport. The genes identified in *P. akamusi* are all marked with black boxes. To enhance readability, all the uptake transporters are placed on the upper part of the cell membrane of these imaginary cells, while all the efflux transporters are positioned on the lower part. The blue-colored font represents genes that are downregulated during Cu stress. The red-colored font represents genes that are upregulated during Cu stress.

In this study, based on the results obtained from ClustalX and Pfam analyses, a total of six ZNT genes were identified in *P. akamusi*, all of which possess six transmembrane domains. In order to verify which subfamily these six genes belong to, we used the ten ZNT genes that have been verified in humans and mice from an NCBI search [104] and ZNT genes of *D. melanogaster* and *P. akamusi* to construct a phylogenetic tree by the M-L method (Figure 5B). The results showed that the Pa ZNT was distributed in all three subgroups. We found that Pa ZNT-4 and Pa ZNT-8 belong to the ZNT III subfamily; Pa ZNT-1, Pa ZNT-10, and Pa ZNT-7 belong to the ZNT II subfamily; and Pa ZNT-9 belongs to the ZNT I subfamily. We did not find the homologous protein of ZNT-6 in *P. akamusi*. Subsequently, we utilized the ClustalX program to align the sequences of the ZnT proteins belonging to the three subfamilies. When compared, like other ZNT proteins, Pa ZNT proteins share a common structure consisting of six transmembrane helices, and Pa ZNT-1, Pa ZNT-4, Pa ZNT-7, and Pa ZNT-8 contain a lengthy histidine-rich loop situated between TMDs IV and V, following the pattern of (Hx)n where n ranges from 3 to 15 (Figure S7). In addition, we found that the homolog protein of ZNT-9 in *P. akamus* aligns well with other ZNT-9s. The alignment of ZNT-9 indicates that ZNT-9 and its corresponding proteins display considerable similarity to both the DNA-associating region and the sequence for nuclear receptor binding. Additionally, ZNT-9 contains a contiguous polypeptide sequence of 10 amino acids, specifically LEVWGSXEAL [105] (Figure S8). The diagram of heavy metal transport in *P. akamusi* is shown in Figure 6.

### 3.5. The Transcriptomic Response to Cu Exposure

In order to study the changes in these enzymes in the hormoone, chitin metabolism, and metal transport pathways in *P. akamusi* exposed to heavy metals, we present the gene expression profiles of fourth-instar larvae of *P. akamusi* following exposure to 20 mg/L of CuSO_4_ solution for 24, 48, 72, and 96 h, with larvae exposed to water serving as the control group. The results indicate that under copper stress, the expression of genes involved in various pathways in *P. akamusi* is generally affected, as detailed below.

**Figure 7:**
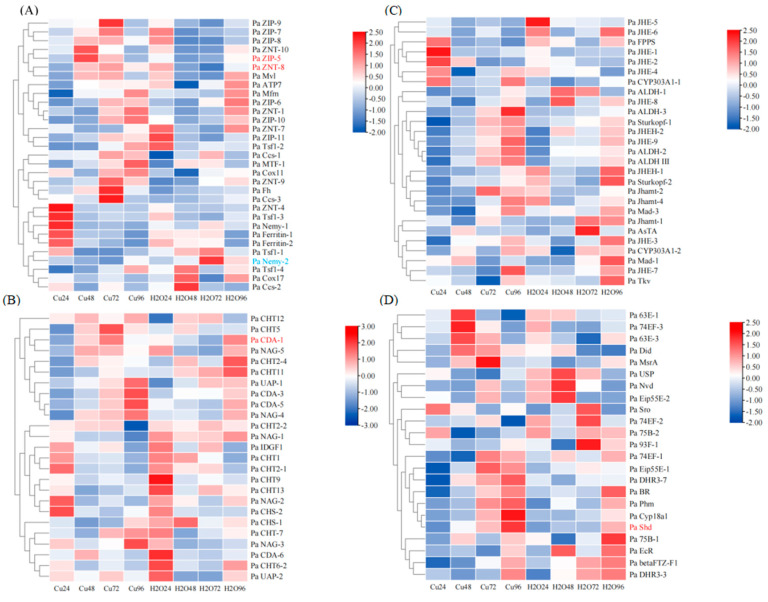
The impact of heavy metal exposure on pupariation and gene expression. Cu stress altered the expression profiles of most genes, with genes exhibiting statistically significant inductions in expression levels denoted in the red font and genes showing statistically significant suppressions in expression levels indicated in the blue font. (**A**) A heatmap illustrating the transcription profiles of genes related to the metal transport pathway in *P. akamusi* across different days with or without treatment with 20 mg/L copper. (**B**) A heatmap depicting the transcription profiles of chitin pathway genes in *P. akamusi* across different days with or without treatment with 20 mg/L copper. The genes shown in the blue font represent those downregulated under copper stress, while those in the red font are upregulated under copper stress. (**C**) A heatmap illustrating the transcription profiles of genes related to the JH pathway in *P. akamusi* across different days with or without treatment with 20 mg/L copper. (**D**) A heatmap illustrating the transcription profiles of genes related to the 20E pathway in *P. akamusi* across different days with or without treatment with 20 mg/L copper.

In the metal transport pathway, the research findings revealed that under heavy metal stress, genes associated with zinc transport were generally suppressed within the first 24 h but were almost entirely induced and upregulated between 48 and 72 h. At 96 h, the expression of nearly half of these genes was suppressed again. Notably, compared to the control group, the expressions of Pa ZIP-5 and Pa ZIP-7 were significantly induced at 48 h. This pattern of expression changes is inversely related to that of MTF-1, which plays a central role in heavy metal homeostasis and detoxification. The expression patterns of genes related to iron transport under copper stress are relatively complex. Among them, the expression of Pa Fh is consistently induced from 24 to 96 h, while Pa Mfrn remains induced after 24 h. Pa Tsf1-2 is induced under long-term stress (96 h), whereas Pa Tsf1-3 shows the opposite trend, being suppressed under long-term stress. The expression changes in the other genes do not exhibit clear trends. Genes associated with copper transport are generally induced at the onset of stress but gradually suppressed thereafter. Notably, Pa Cox11 is induced throughout the entire phase of copper stress. It is also worth mentioning that Pa Nemy-2 is significantly suppressed at 72 h (Figure 7A).

In the chitin synthesis metabolic pathway, although the expression of genes related to chitin synthesis was suppressed at 72 h, they generally showed a trend of being induced, such as with Pa UAP-1 and Pa CHS-2. This suggests that heavy metal stress may accelerate chitin synthesis in *P. akamusi*. In contrast, under copper stress, the expression patterns of genes related to chitin degradation are relatively complex. According to Figure 7B, they can be roughly categorized into two groups. The nine degradation-related genes ranging from Pa CHT12 to Pa NAG-4 generally exhibited suppressed expression within the first 24 h, followed by gradual induction. On the other hand, the 12 degradation-related genes from Pa CHT2-2 to Pa UAP-2 were predominantly suppressed in their overall expression. Notably, Pa CDA-1 was significantly induced at 72 h (Figure 7B).

In the JH metabolic pathway, genes associated with JH synthesis were generally suppressed in expression during the first 48 h of Cu stress and thereafter they were gradually induced. Among them, the expression of Pa 303A1-1 was consistently induced throughout this stress period. Genes related to JH degradation were induced at the initial stage of stress (24 h) but generally suppressed between 48 and 72 h, followed by gradual induction thereafter. Notably, the expressions of Pa JHE-6, Pa JHEH-1, and the physiologically functional JHE-5 gene were continuously suppressed throughout the entire stress process. In comparison to the control group, the expression patterns of other JH-related genes under copper stress were relatively complex (Figure 7C).

In the metabolic pathway of 20E, the majority of genes associated with 20E synthesis were suppressed during the first 24 h of Cu exposure and gradually induced thereafter, becoming universally upregulated by 96 h. Notably, Pa Shd-2 was significantly induced at 72 h. Heavy metal exposure accelerated 20E synthesis. Concurrently, Pa CYP18a1, a gene related to 20E degradation, was suppressed during the first 24 h and continuously induced thereafter, suggesting that heavy metal stress accelerated 20E degradation in *P. akamusi*. Although the suppression of expression for other genes related to 20E slightly recovered at 24 and 76 h, they were generally suppressed overall (Figure 7D).

## 4. Discussion

Cuticles function as the exoskeletons of insects, playing a pivotal role in determining their physiological development and resilience to environmental stressors. Chitin constitutes a primary component of this cuticle. In order to accommodate growth, insects must be repeatedly shed and regrow their chitin exoskeleton during molting, a process tightly regulated by 20E and JH. However, there has been no systematic annotation of JH and 20E, and previous studies have not investigated the related genes across species within the Chironomidae family. In this study, we annotated genes associated with JH, 20E, and metal transport in *P. akamusi* based on a previously assembled genome sequence. A total of 139 genes were identified, with 109 of them clearly classifiable into three distinct metabolic pathways: 27 genes involved in the JH metabolic pathway, 24 in the 20E metabolic pathway, 27 in the chitin metabolic pathway, and 31 related to metal transport. Among several key enzymes, we identified specific classical consensus motifs and conserved domains unique to each gene family, and these were all highly consistent with the conserved regions of related genes in species such as *D. melanogaster* and *An. gambiae*. Furthermore, phylogenetic analysis and classification were conducted for genes such as JHE, CHT, ZIP, and ZNT. These genes could be accurately categorized into their respective subfamilies, possessing conserved sequence characteristics typical of each subfamily. The remaining 30 genes failed to pass Pfam validation and lacked conserved motifs associated with relevant genes, and thus they were temporarily excluded.

Additionally, we observed that under Cu stress, the expression profiles of most genes associated with JH, 20E, chitin, and metal transport were altered, exhibiting complex fluctuations over long-term exposure. Meanwhile, different members of the same gene family, such as JHE and CHT, responded differently to the same stress, including upregulation, downregulation, and showing no change.

Specifically, in the heavy metal transport pathway, the expression of Pa ZNT-5 and Pa ZIP-7 were significantly affected. Zinc ions are crucial for MTF-1, which plays a pivotal role in controlling cellular detoxification. Given that the expression of genes associated with zinc ion concentration was significantly impacted, this suggests that, under copper stress, *P. akamusi* may primarily respond to heavy metal stress through the transport of zinc ions. The homeostasis of Fe and Cu is crucial for maintaining normal physiological activities in the body, such as redox reactions, electron transfer, oxygen transport, and energy metabolism. Imbalances in intracellular Fe and Cu can lead to DNA double-strand breaks, cell cycle arrest, mitochondrial dysfunction, lipid peroxidation, and protein modification, ultimately resulting in cell death. Additionally, both iron and copper can induce ferroptosis. Except for Pa Nemy-2, the expression of other genes related to iron and copper transport was not significantly affected.

In the chitin synthesis metabolic pathway, the expression of Pa CDA-1 was significantly affected, indicating that this gene may be the primary responder associated with chitin degradation in *P. akamusi* under heavy metal stress. The expression patterns of other genes related to chitin synthesis and degradation are relatively complex, with different genes within the same family exhibiting distinct expression patterns. It is possible that, under copper stress, these genes perform different functions to cope with the impact of copper on the normal synthesis and degradation of chitin in the *P. akamusi*.

In the JH metabolic pathway, Pa JHE-5 is a JH-degrading enzyme that exerts physiological functions in *P. akamusi*, and its expression was persistently and significantly suppressed under heavy metal stress, suggesting that heavy metals may reduce the degradation rate of JH. The changes in several other genes were more complex. Overall, prolonged exposure to heavy metals may inhibit JH degradation, leading to an increase in JH content within *P. akamusi*. This could potentially cause the larvae of *P. akamusi* to remain in the molting stage, thereby altering their molting cycle and rhythm.

In the 20E metabolic pathway, the expression of Pa Phm-1 was significantly affected. Overall, under Cu stress, genes related to both 20E synthesis and degradation were induced, suggesting that heavy metal exposure accelerated 20E synthesis and metabolism in *P. akamusi*, thereby altering the molting rate and affecting the normal growth and development of *P. akamusi*.

Under copper stress, the expression change curves of genes with relatively prominent variations in each pathway are illustrated in Figure S9. Among them, the expression changes in Pa Shd, Pa CDA-1, Pa nemy-2, Pa ZIP-5, and Pa ZIP-7 are statistically significant. These genes are likely to be response genes of *P. akamusi*, modulating in response to heavy metal stress, playing crucial roles in the molting process and detoxification metabolism of *P. akamusi* larvae. They can serve as potential target genes for future RNAi studies, which may help to improve the silencing efficiency of RNAi. However, these potential response genes are preliminary results obtained using relaxed criteria, and further validation is required in subsequent research. Based on gene functional annotations and relevant research analysis results, among the genes with relatively prominent expression changes, genes such as CDA, NAG, FPPS, Shd, Phm, JHAMT, and CHT participate in specific pathways [58,106,107,108,109,110,111]. Mad [112], 63E, betaFTZ-F1-1 [113], DHR3-3 [114], and USP [115] are mostly related to insect hormone signaling pathways and developmental regulation. Therefore, the expression changes in these genes are more inclined to be a matter of pathway-specific regulation. ALDH plays a key role in cellular detoxification processes and oxidative stress responses [116]. Ccs may be expressed under various heavy metal stresses and is associated with heat stress [117]. These two gene changes are likely the result of general stress responses.

Our study provides new insights into insect toxicology research and advances the study of detoxification mechanisms and the regulation of growth and development in insects. However, there are still some limitations. Firstly, this study primarily focuses on gene-level analysis. Further functional validation studies are required to confirm the roles of these genes. For the potential response genes, we did not verify their correlations at the gene expression level through methods such as quantitative real-time polymerase chain reaction (qPCR). In addition, our deficiencies in continuous generation culture techniques and the efficiency of RNAi technology have directly impacted the depth and breadth of our research.

In the future, we plan to conduct an orthogonal validation of the response genes, seek advice, and learn from experts who have mastered the continuous generation culture techniques of chironomids. Moreover, we will attempt to use different RNAi methods and optimize the experimental design of RNAi to improve gene silencing efficiency. We also intend to validate these genes in other species of the Chironomidae family. This will contribute to a comprehensive and in-depth understanding of the complex genetic regulatory mechanisms underlying the molting process in Chironomidae species, including *P. akamusi*, and promote research progress in the field of insect toxicology.

## Data Availability

The original contributions presented in this study are included in the Article/Supplementary Materials. Further inquiries can be directed to the corresponding author.

## Supplementary Materials

The following supporting information can be downloaded at: https://www.mdpi.com/article/10.3390/insects16060636/s1, Table S1: Accession numbers of proteins used in the paper. Table S2: Raw base. Table S3: Gene expression levels. Figure S1: Amino acid sequence alignment of *P. akamusi*, *D. melanogaster*, *H. sapiens*, and *M. musculus* CG275 and Nemy. Figure S2: Amino acid sequence alignment of *P. akamusi*, *D. melanogaster*, and *An. gambiae* Ferritin. Figure S3: Amino acid sequence alignment of *P. akamusi*, *D. melanogaster*, and *An. gambiae* Cox17. Figure S4: Amino acid sequence alignment of *P. akamusi*, *D. melanogaster*, and *An. gambiae* ATP7. Figure S5: The TMIV, TMV, and HExPHExGD motifs for the LIV/LZT subfamilies of *P. akamusi*, *D. melanogaster*, *An. gambiae*, *Homo sapiens*, and *Mus musculus*. Figure S6: The TMIII, TMIV, and conserved Lx(2)Hx(4)GxAxG and HKx(4)F motifs for the ZIPI and ZIPII subfamilies of *P. akamusi*, *Homo sapiens*, *D. melanogaster*, *Mus musculus*, and *An. gambiae*. Figure S7: The alignment of the TMIV, TMV, and His-rich loop of ZNTs among *P. akamusi*. Figure S8: (C) The alignment of the conserved LEVWGSXEAL motifs of the ZNTI subfamily among *P. akamusi*, *Homo sapiens*, *D. melanogaster*, *Mus musculus*, and *An. gambiae*. Figure S9: Line graph of primary responsive gene expression levels.
