# Supplementary material for: Genome Annotation of Molting-Related Protein-Coding Genes in Propsilocerus akamusi Reveals Transcriptomic Responses to Heavy Metal Contamination
Source: Insects. 2025 Jun 17;16(6):636. doi: 10.3390/insects16060636 (PMC12193260; doi:10.3390/insects16060636)
Supplement: Supplementary file 1 [file insects-16-00636-s001.zip › Figure caption.pdf]

Figure S1: Amino acid sequence alignment of *P. akamusi*, *D. melanogaster*, *H. sapiens* and *M. musculus* CG275, Nemy.

Figure S2: Amino acid sequence alignment of *P. akamusi*, *D. melanogaster* and *An. gambiae* Ferritin.

Figure S3: Amino acid sequence alignment of *P. akamusi*, *D. melanogaster* and *An. gambiae* Cox17.

Figure S4: Amino acid sequence alignment of *P. akamusi*, *D. melanogaster* and *An. gambiae* ATP7.

Figure S5: The TMIV , TMV and HExPHExGD motifs for LIV/LZT subfamilies of *P. akamusi*, *D. melanogaster*, *An. gambiae, Homo sapiens* and *Mus musculus*.

Figure S6: The TMIII , TMIV and the conserved Lx(2)Hx(4)GxAxG and HKx(4)F motifs for ZIPI and ZIPII subfamilies of *P. akamusi*, *Homo sapiens*, *D. melanogaster*, *Mus musculus* and *An. gambiae*.

Figure S7: The alignment of the TM IV , TM V and His-rich loop of ZNTs among *P. akamusi*.

Figure S8: (C) The alignment of the conserved LEVWGSXEAL motifs of ZNT I subfamilies among *P. akamusi*, *Homo sapiens*, *D. melanogaster*, *Mus musculus* and *An. gambiae*.

Figure S9: The line graph of primary responsive gene expression levels.
