# Supplementary material for: Genome Annotation of Molting-Related Protein-Coding Genes in Propsilocerus akamusi Reveals Transcriptomic Responses to Heavy Metal Contamination
Source: Insects. 2025 Jun 17;16(6):636. doi: 10.3390/insects16060636 (PMC12193260; doi:10.3390/insects16060636)
Supplement: Supplementary file 1 [file insects-16-00636-s001.zip › Figure S2.pdf]

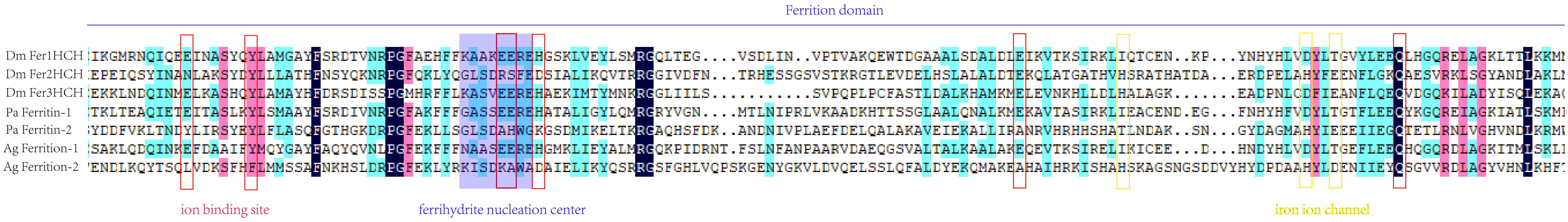

Ferrition domain
Dm Fer1HCH
Dm Fer2HCH
Dm Fer3HCH
Pa Ferritin-1
Pa Ferritin-2
Ag Ferrition-1
Ag Ferrition-2
ion binding site
ferrihydrite nucleation center
iron ion channel
