# Supplementary material for: Genome Annotation of Molting-Related Protein-Coding Genes in Propsilocerus akamusi Reveals Transcriptomic Responses to Heavy Metal Contamination
Source: Insects. 2025 Jun 17;16(6):636. doi: 10.3390/insects16060636 (PMC12193260; doi:10.3390/insects16060636)
Supplement: Supplementary file 1 [file insects-16-00636-s001.zip › Figure S3.pdf]

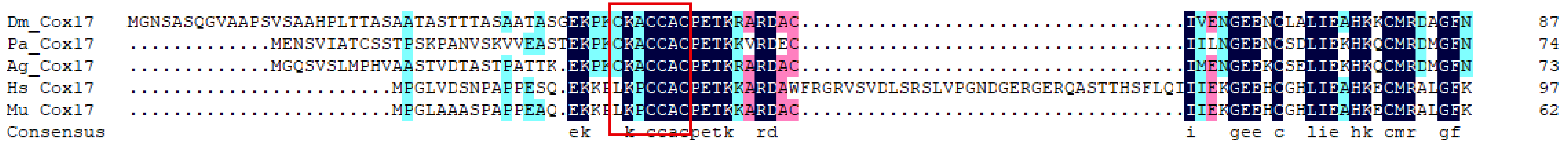

Dm_Cox17 MGNSASQGVAAPSVSAAHPLTTASAATASTTTASAATASGEKPKCKACCACPETKRARDAC...................................IVENGEENCLALIEAHKKCMRDAGFN 87
Pa_Cox17 .............MENSVIATCSSTPSKPANVSKVVEASTEKPKCKACCACPETKKVRDEC...................................IILNGEENCSDLIEKHKQCMRDMGFN 74
Ag_Cox17 .............MGQSVSLMPHVAASTVDTASTPATTK.EKPKCKACCACPETKRARDAC...................................IMENGEEKCSELIEKHKQCMRDMGFN 73
Hs_Cox17 ........................MPGLVDSNPAPPESQ.EKKPLKPCCACPETKKARDAWFRGRVSVDLSRSLVPGNDGERGERQASTTHSFLQIIIEKGEEHCGHLIEAHKECMRALGFK 97
Mu_Cox17 ........................MPGLAAASPAPPEAQ.EKKPLKPCCACPETKKARDAC...................................IIEKGEEHCGHLIEAHKECMRALGFK 62
Consensus ek k ccacpetk rd i gee c lie hk cmr gf
