# Supplementary material for: Genome Annotation of Molting-Related Protein-Coding Genes in Propsilocerus akamusi Reveals Transcriptomic Responses to Heavy Metal Contamination
Source: Insects. 2025 Jun 17;16(6):636. doi: 10.3390/insects16060636 (PMC12193260; doi:10.3390/insects16060636)
Supplement: Supplementary file 1 [file insects-16-00636-s001.zip › Figure S7.pdf]

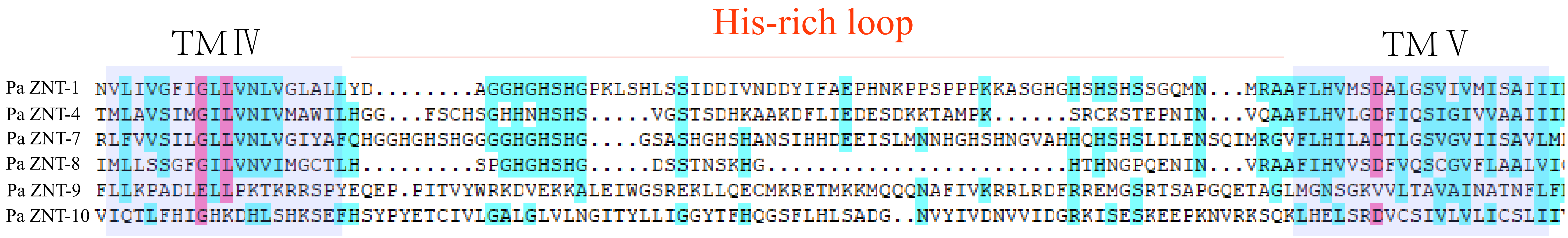
His-rich loop
TM Ⅳ
TM Ⅴ
Pa ZNT-1
Pa ZNT-4
Pa ZNT-7
Pa ZNT-8
Pa ZNT-9
Pa ZNT-10
