# Supplementary material for: Genome Annotation of Molting-Related Protein-Coding Genes in Propsilocerus akamusi Reveals Transcriptomic Responses to Heavy Metal Contamination
Source: Insects. 2025 Jun 17;16(6):636. doi: 10.3390/insects16060636 (PMC12193260; doi:10.3390/insects16060636)
Supplement: Supplementary file 1 [file insects-16-00636-s001.zip › Figure S8.pdf]

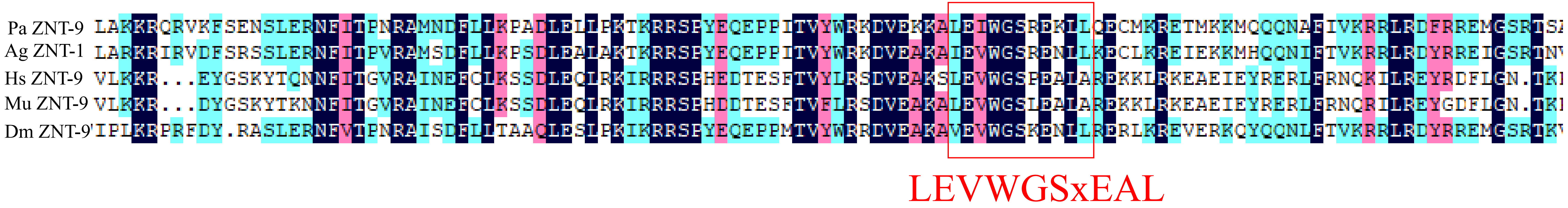
Pa ZNT-9 LAKKRQRVKFSENSLERNFITPNRAMNDFLLKPADLELLPKTKRRSPYEQEPPITVYWRKDVEKKALEIWGSREKLLQECMKRETMKKMQQQNAFIVKRRLRDFRREMGSRTS
Ag ZNT-1 LARKRIRVDFSRSSLERNFITPVRAMSDFLLKPSDLEALAKTKRRSPYEQEPPITVYWRKDVEAKAIEVWGSRENLLKECLKREIEKKMHQQNIFTVKRRLRDYRREIGSRTN
Hs ZNT-9 VLKKR...EYGSKYTQNNFITGVRAINEFCLKSSDLEQLRKIRRRSPHEDTESFTVYLRSDVEAKSLEVWGSPEALAREKKLRKEAEIEYRERLFRNQKILREYRDFLGN.TK
Mu ZNT-9 VLKKR...DYGSKYTKNNFITGVRAINEFCLKSSDLEQLRKIRRRSPHDDTESFTVFLRSDVEAKALEVWGSLEALAREKKLRKEAEIEYRERLFRNQRILREYGDFLGN.TK
Dm ZNT-9'IPLKRPRFDY.RASLERNFVTPNRAISDFLLTAAQLESLPKIKRRSPYEQEPPMTVYWRRDVEAKAVEVWGSKENLLRERLKREVERKQYQQNLFTVKRRLRDYRREMGSRTK
LEVWGSxEAL
