# Supplementary material for: Genome Annotation of Molting-Related Protein-Coding Genes in Propsilocerus akamusi Reveals Transcriptomic Responses to Heavy Metal Contamination
Source: Insects. 2025 Jun 17;16(6):636. doi: 10.3390/insects16060636 (PMC12193260; doi:10.3390/insects16060636)
Supplement: Supplementary file 1 [file insects-16-00636-s001.zip › Figure S9.pdf]

20E metabolic pathway

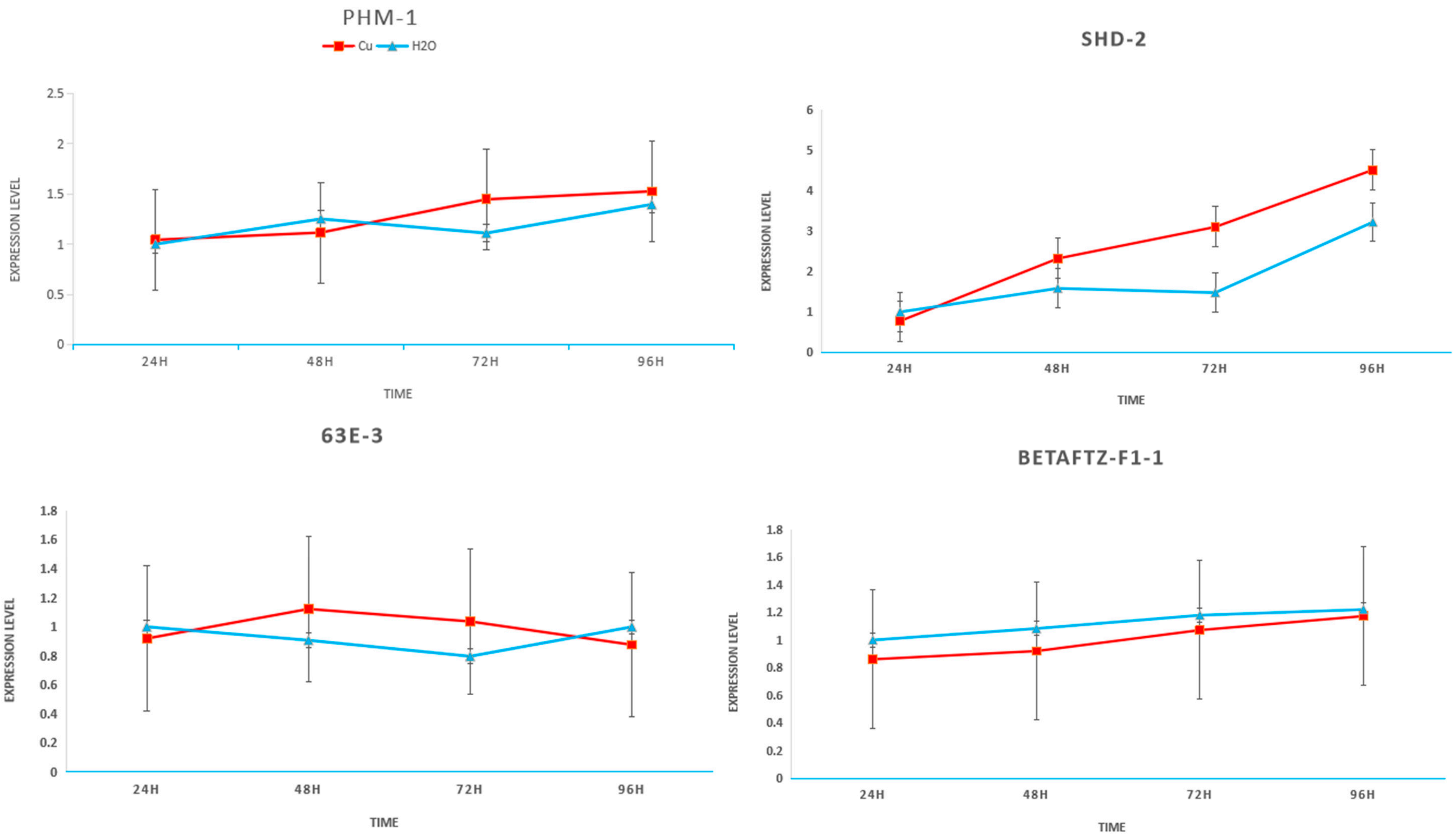

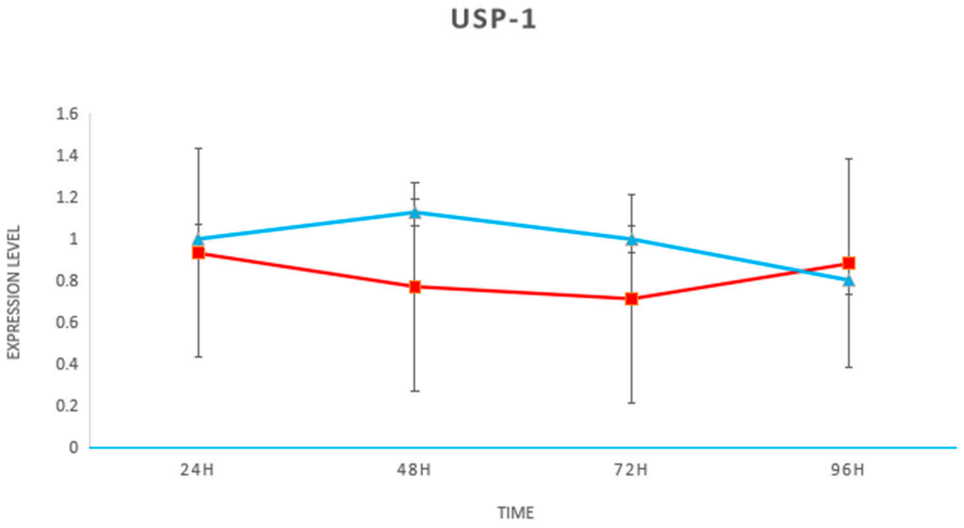
USP-1
EXPRESSION LEVEL
1.6
1.4
1.2
1
0.8
0.6
0.4
0.2
0
24H
48H
72H
96H
TIME

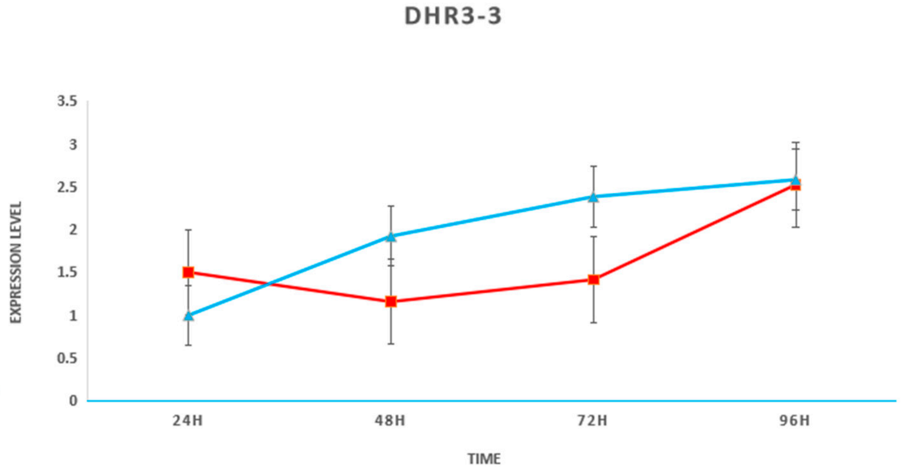
DHR3-3
EXPRESSION LEVEL
3.5
3
2.5
2
1.5
1
0.5
0
24H
48H
72H
96H
TIME

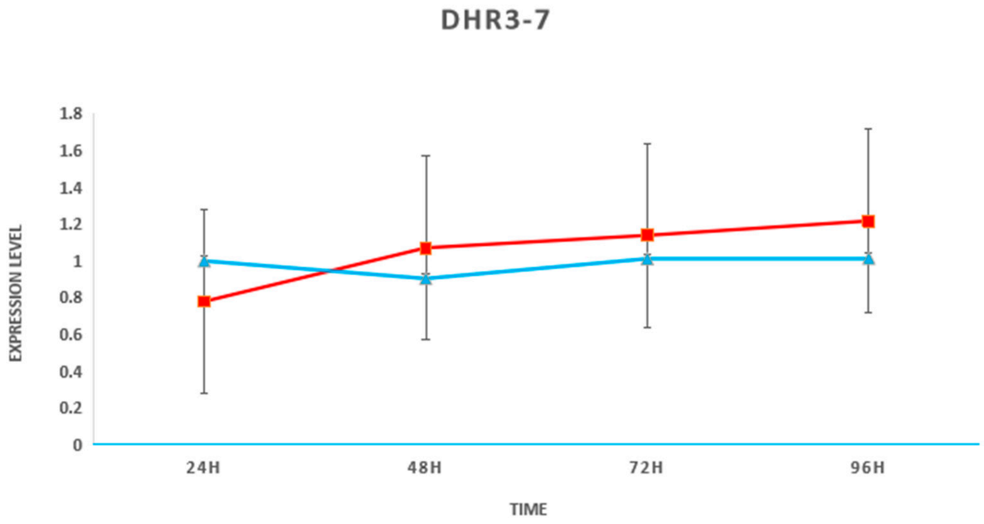
DHR3-7
EXPRESSION LEVEL
1.8
1.6
1.4
1.2
1
0.8
0.6
0.4
0.2
0
24H
48H
72H
96H
TIME

JH metabolic pathway

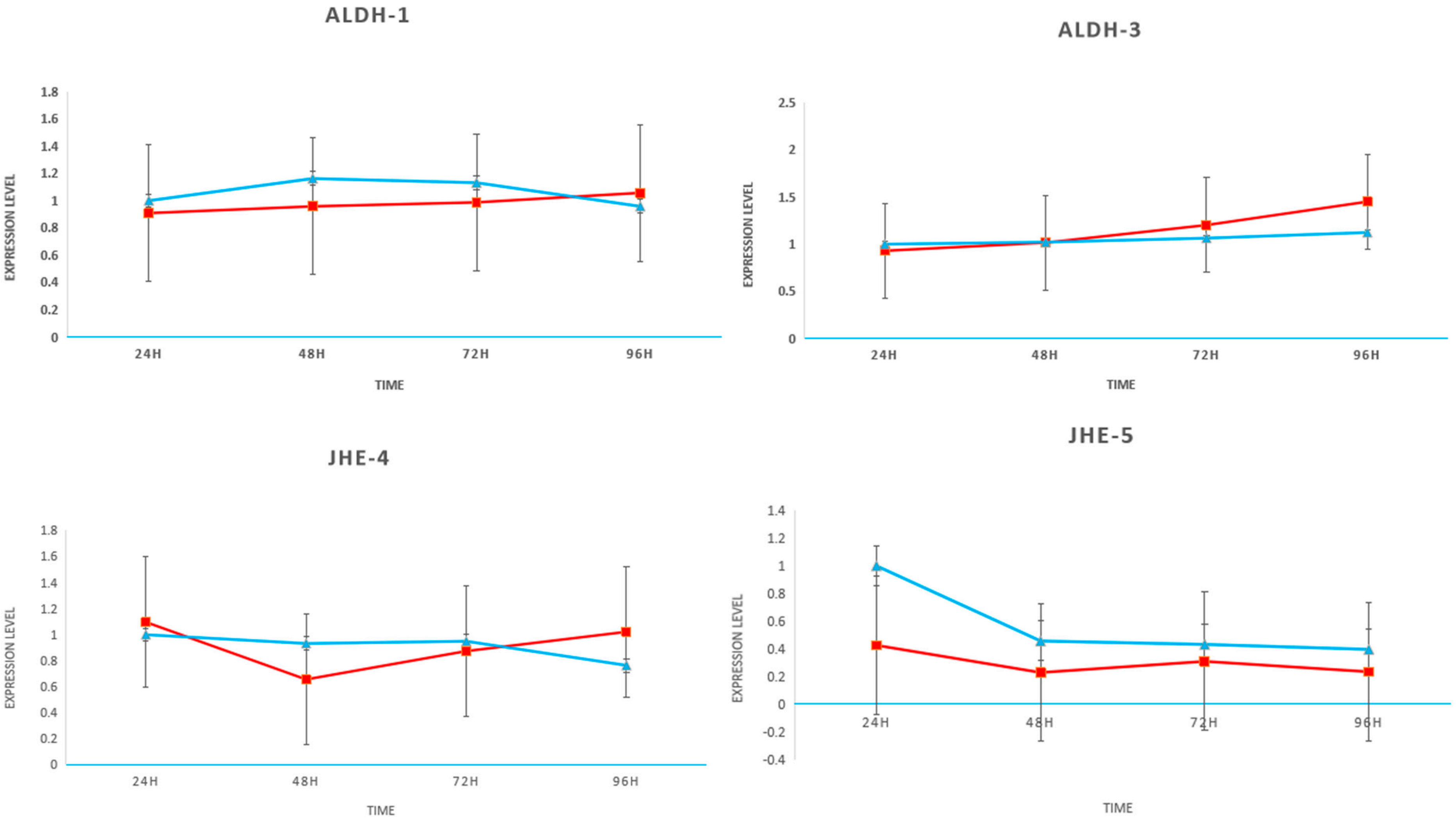

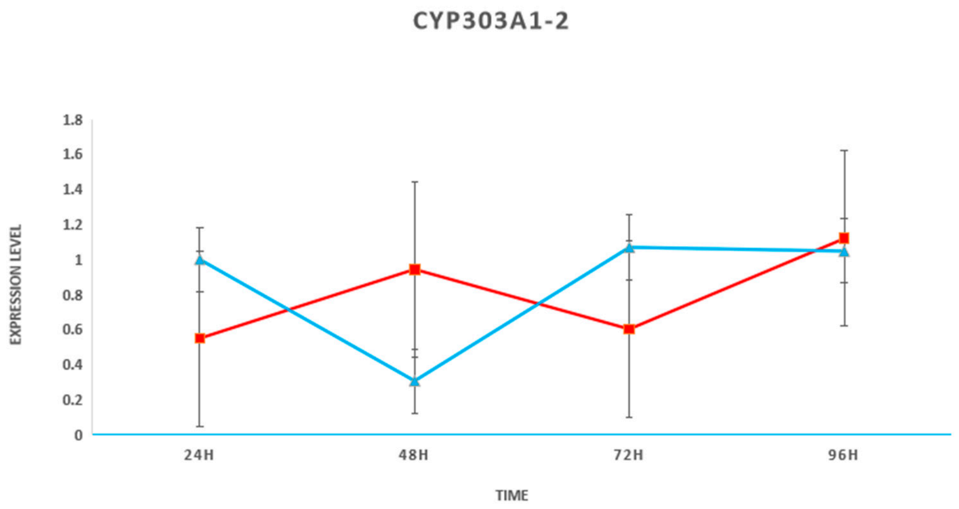
CYP303A1-2
EXPRESSION LEVEL
TIME
24H
48H
72H
96H

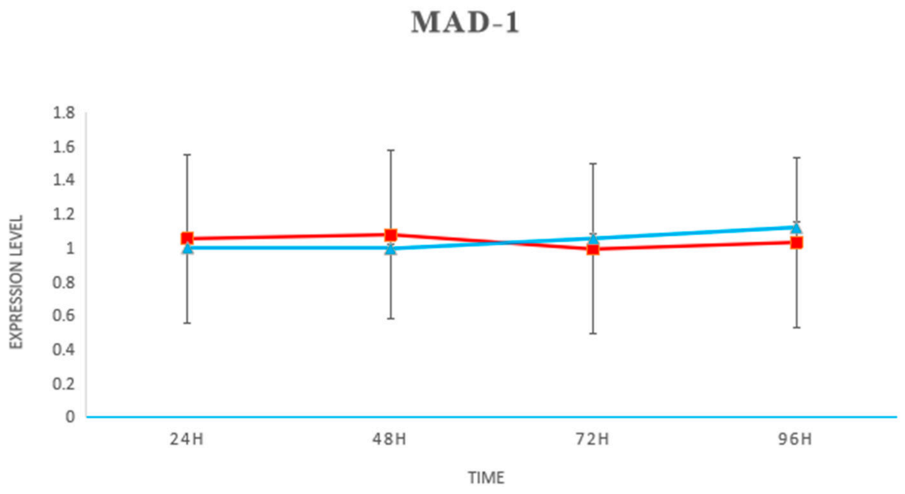
MAD-1
EXPRESSION LEVEL
TIME
24H
48H
72H
96H

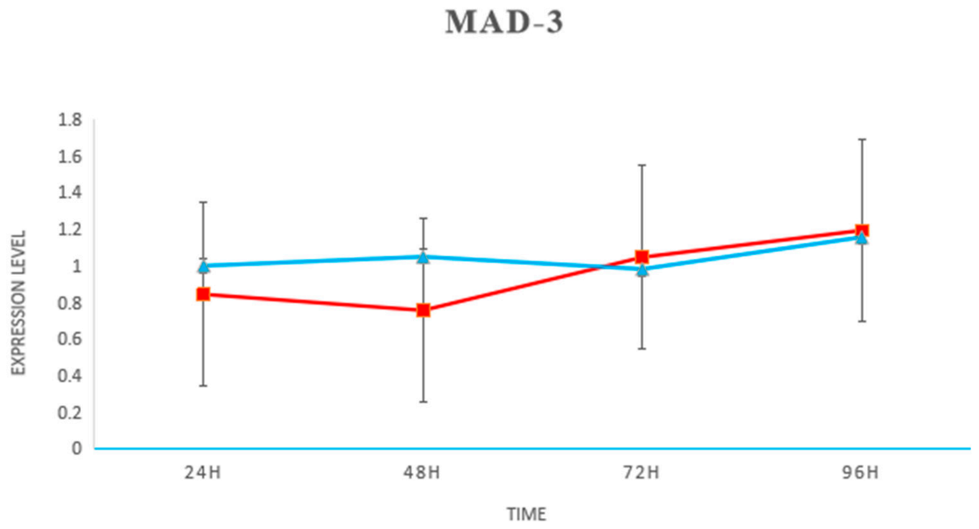
MAD-3
EXPRESSION LEVEL
TIME
24H
48H
72H
96H

Chitin metabolic pathway

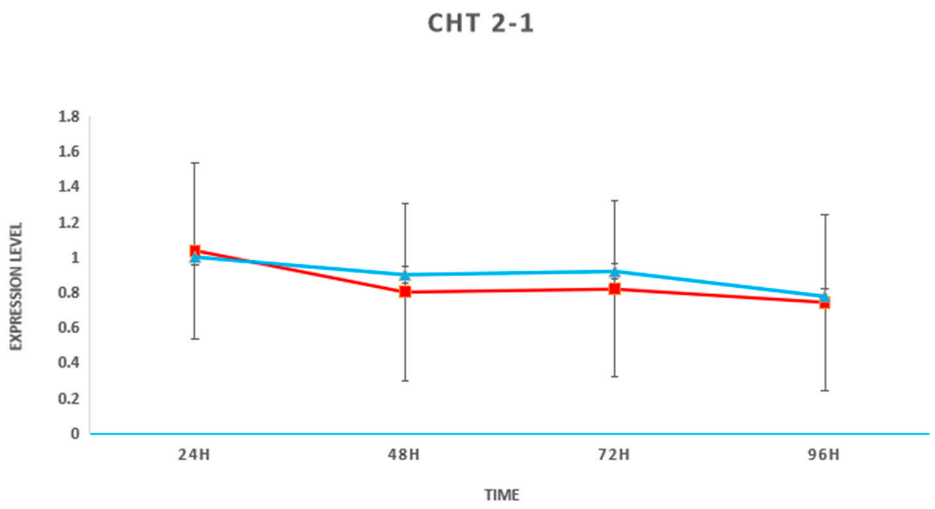

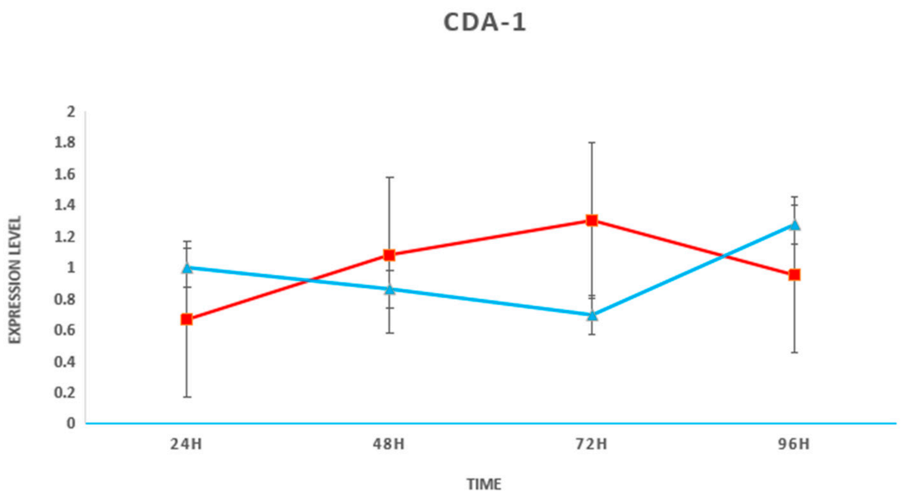

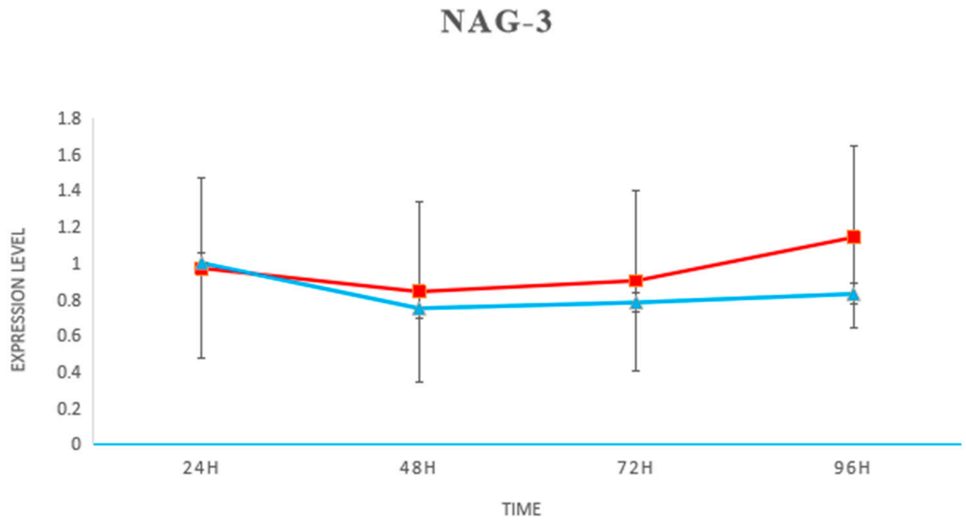

Metal transport pathway

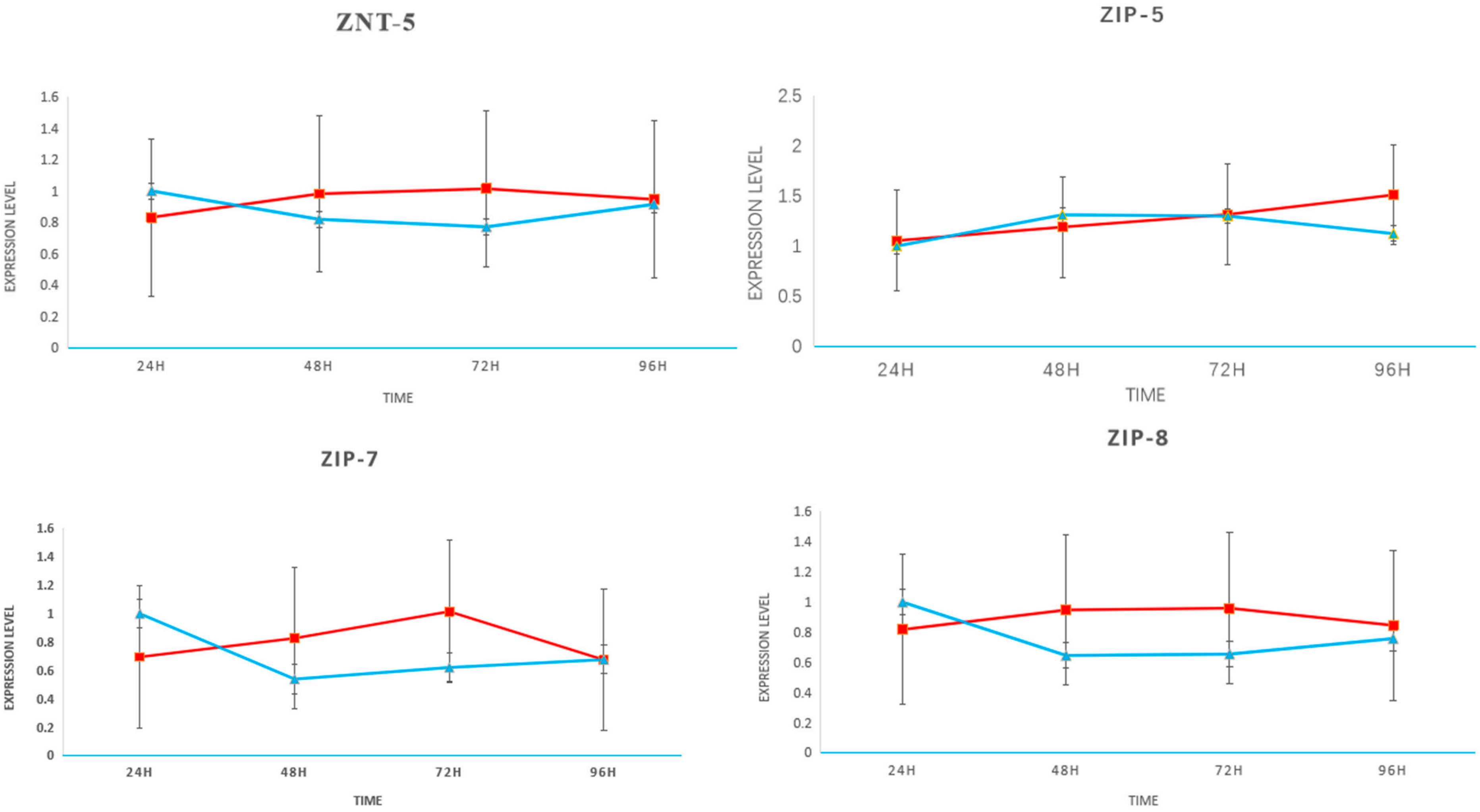

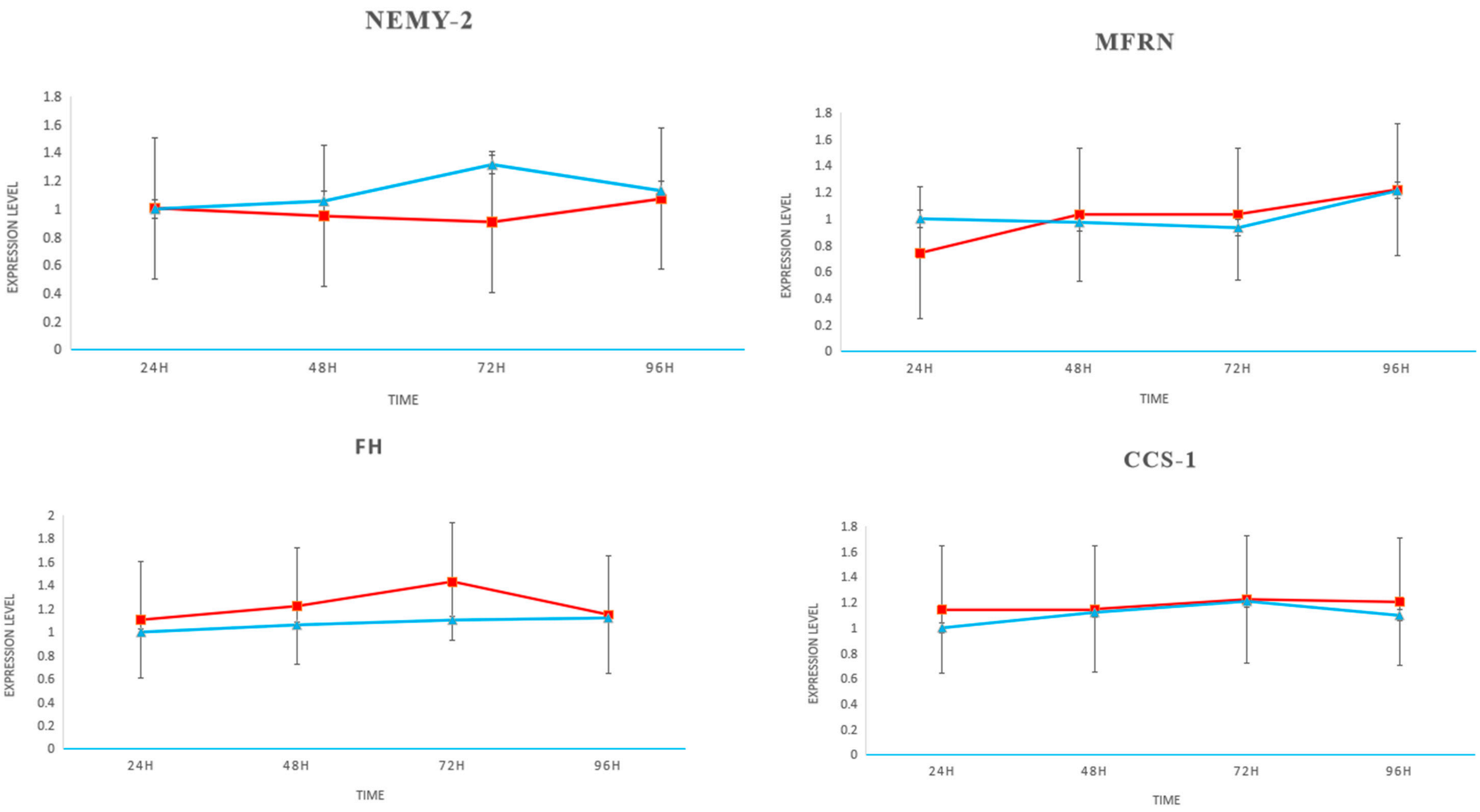
NEMY-2
EXPRESSION LEVEL
0
0.2
0.4
0.6
0.8
1
1.2
1.4
1.6
1.8
24H
48H
72H
96H
TIME
MFRN
EXPRESSION LEVEL
0
0.2
0.4
0.6
0.8
1
1.2
1.4
1.6
1.8
24H
48H
72H
96H
TIME
FH
EXPRESSION LEVEL
0
0.2
0.4
0.6
0.8
1
1.2
1.4
1.6
1.8
2
24H
48H
72H
96H
TIME
CCS-1
EXPRESSION LEVEL
0
0.2
0.4
0.6
0.8
1
1.2
1.4
1.6
1.8
24H
48H
72H
96H
TIME

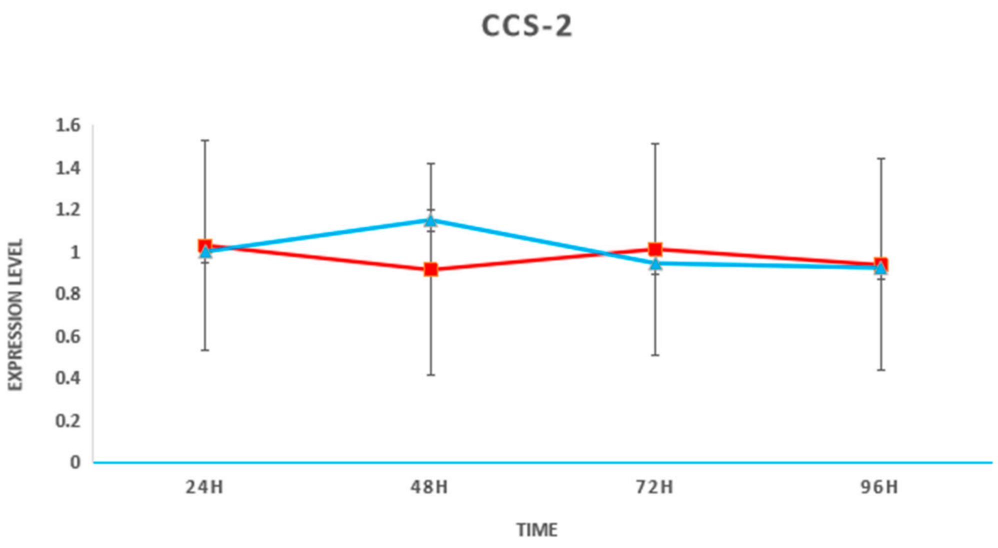
CCS-2
EXPRESSION LEVEL
1.6
1.4
1.2
1
0.8
0.6
0.4
0.2
0
24H
48H
72H
96H
TIME
