# Supplementary material for: Genome Annotation of Molting-Related Protein-Coding Genes in Propsilocerus akamusi Reveals Transcriptomic Responses to Heavy Metal Contamination
Source: Insects. 2025 Jun 17;16(6):636. doi: 10.3390/insects16060636 (PMC12193260; doi:10.3390/insects16060636)
Supplement: Supplementary file 1 [file insects-16-00636-s001.zip › Table S1.pdf]

Table S1. Assession numbers of proteins used in Paper.

Name in Paper	Species	Accession
Dm Nvd	*Drosophila melanogaster*	NP_001097670.1
Dm Spo	*Drosophila melanogaster*	NP_001286943.1
Dm Sro	*Drosophila melanogaster*	NP_651725.1
Dm Phm	*Drosophila melanogaster*	NP_001285414.1
Dm Cyp18a1	*Drosophila melanogaster*	NP_523403.2
Dm Did	*Drosophila melanogaster*	NP_524810.2
Dm Shd	*Drosophila melanogaster*	NP_001261843.1
Dm Eip55E	*Drosophila melanogaster*	NP_001286578.1
Dm 74EF	*Drosophila melanogaster*	NP_001014590.1
Dm 75B	*Drosophila melanogaster*	NP_001246822.1
Dm 63E	*Drosophila melanogaster*	NP_001189042.1
Dm 93F	*Drosophila melanogaster*	NP_001097865.1
Dm MsrA	*Drosophila melanogaster*	NP_730048.1
Dm EcR	*Drosophila melanogaster*	NP_730048.1
Dm BR	*Drosophila melanogaster*	NP_001188527.1

Dm betaFTZ-F1	*Drosophila melanogaster*	NP_001246824.1
Dm DHR3(HR3)	*Drosophila melanogaster*	NP_001334718.1
Dm USP	*Drosophila melanogaster*	NP_001259168.1
Dm FPPS	*Drosophila melanogaster*	NP_477380.1
Dm ALDH	*Drosophila melanogaster*	NP_609285.1
Dm ALDHIII	*Drosophila melanogaster*	NP_724565.3
Dm Jhamt	*Drosophila melanogaster*	NP_001285980.1
Dm JHE	*Drosophila melanogaster*	NP_523758.3
Dm JHEH1	*Drosophila melanogaster*	NP_611385.1
Dm JHEH2	*Drosophila melanogaster*	NP_788413.4
Dm CYP303A1	*Drosophila melanogaster*	NP_001285977.1
Dm Mad	*Drosophila melanogaster*	NP_001259992.1
Dm Mip	*Drosophila melanogaster*	NP_648971.1
Dm AstC	*Drosophila melanogaster*	NP_001162948.1
Dm AsTA	*Drosophila melanogaster*	NP_524489.2
Dm Sturkopf	*Drosophila melanogaster*	NP_612097.1
Dm CDA-1	*Drosophila melanogaster*	NP_728468.1
Dm CDA-2	*Drosophila melanogaster*	NP_001245806.1

Dm CDA-3	*Drosophila melanogaster*	NP_001286519.1
Dm CDA-4	*Drosophila melanogaster*	NP_609806.1
Dm CDA-5	*Drosophila melanogaster*	NP_730444.1
Dm CDA-6	*Drosophila melanogaster*	NP_730442.2
Dm CHT-1	*Drosophila melanogaster*	NP_609190.2
Dm CHT-2	*Drosophila melanogaster*	NP_477298.2
Dm CHT-4	*Drosophila melanogaster*	NP_524962.2
Dm CHT-5	*Drosophila melanogaster*	NP_650314.1
Dm CHT-6	*Drosophila melanogaster*	NP_572598.3
Dm CHT-7	*Drosophila melanogaster*	NP_647768.3
Dm CHT-8	*Drosophila melanogaster*	NP_611542.2
Dm CHT-9	*Drosophila melanogaster*	NP_611543.3
Dm CHT-10	*Drosophila melanogaster*	EAA46011.1
Dm CHT-11	*Drosophila melanogaster*	NP_572361.1
Dm CHT-12	*Drosophila melanogaster*	NP_726022.1
Dm IDGF1	*Drosophila melanogaster*	NP_477258.1
Dm IDGF2	*Drosophila melanogaster*	NP_477257.2
Dm IDGF3	*Drosophila melanogaster*	NP_723967.1

Dm IDGF4	*Drosophila melanogaster*	NP_727374.1
Dm IDGF5	*Drosophila melanogaster*	NP_611321.3
Dm IDGF6	*Drosophila melanogaster*	NP_477081.1
Dm NAG-1	*Drosophila melanogaster*	NP_525081.1
Dm NAG-2	*Drosophila melanogaster*	NP_728975.1
Dm UAP	*Drosophila melanogaster*	NP_001285673.1
Dm UAP	*Drosophila melanogaster*	NP_609032.1
Dm CHS-1	*Drosophila melanogaster*	NP_730928.2
Dm CHS-2	*Drosophila melanogaster*	NP_524209.3
Dm ZNT-1	*Drosophila melanogaster*	NP_647801.1
Dm ZNT-2	*Drosophila melanogaster*	NP_609741.1
Dm ZNT-4	*Drosophila melanogaster*	NP_610185.1
Dm ZNT-7	*Drosophila melanogaster*	NP_650049.1
Dm ZNT-8	*Drosophila melanogaster*	NP_723732.2
Dm ZNT-9	*Drosophila melanogaster*	NP_610806.2
Dm ZNT-10	*Drosophila melanogaster*	NP_649233.2
Dm ZIP1	*Drosophila melanogaster*	Q9V3A4.1
Dm ZIP2	*Drosophila melanogaster*	NP_650440.2

Dm ZIP3	*Drosophila melanogaster*	CAC14874.1
Dm ZIP4	*Drosophila melanogaster*	NP_648732.1
Dm ZIP7	*Drosophila melanogaster*	Q9V3A4.1
Dm ZIP9	*Drosophila melanogaster*	NP_651919.3
Dm ZIP10	*Drosophila melanogaster*	Q9VSL7.3
Dm ZIP11	*Drosophila melanogaster*	NP_610712.1
Dm ZIP13	*Drosophila melanogaster*	Q9VAF0.1
Dm Mvl	*Drosophila melanogaster*	NP_732584.1
Dm mfrn	*Drosophila melanogaster*	NP_651600.1
Dm fh	*Drosophila melanogaster*	NP_511094.1
Dm Fer1	*Drosophila melanogaster*	NP_524873.1
Dm Fer2	*Drosophila melanogaster*	NP_524802.2
Dm Fer3	*Drosophila melanogaster*	NP_572854.1
Dm Tsf-1	*Drosophila melanogaster*	NP_001285404.1
Dm Cox17	*Drosophila melanogaster*	NP_572998.1
Dm Nemy	*Drosophila melanogaster*	NP_725208.1
Dm Ccs	*Drosophila melanogaster*	NP_652029.3
Dm Cox11	*Drosophila melanogaster*	NP_723086.1

Dm ATP7	*Drosophila melanogaster*	NP_001259466.1
Dm MTF-1	*Drosophila melanogaster*	NP_001287002.1
Ag Nvd	*Anopheles gambiae*	XP_309236.5
Ag Phm	*Anopheles gambiae*	ABU42522.1
Ag Did	*Anopheles gambiae*	ABU42523.1
Ag Shd	*Anopheles gambiae*	ABU42525.1
Ag Fpps	*Anopheles gambiae*	EAA04004.4
Ag ALDH	*Anopheles gambiae*	EAA12076.3
Ag Jhamt	*Anopheles gambiae*	XP_314173.1
Ag JHE-1	*Anopheles gambiae*	XP_061513477.1
Ag JHE-2	*Anopheles gambiae*	XP_321363.5
Ag JHE-3	*Anopheles gambiae*	XP_061514545.1
Ag JHE-4	*Anopheles gambiae*	EAU76511.2
Ag JHE-5	*Anopheles gambiae*	EAA11381.4
Ag JHE-8	*Anopheles gambiae*	EAL39528.3
Ag JHE-9	*Anopheles gambiae*	XP_061502644.1
Ag JHE-11	*Anopheles gambiae*	XP_061512604.1
Ag JHE-13	*Anopheles gambiae*	EAA04261.5

Ag JHE-14	*Anopheles gambiae*	EAA13084.4
Ag JHE-15	*Anopheles gambiae*	XP_061513478.1
Ag JHEH	*Anopheles gambiae*	EAL40266.2
Ag CHT-2	*Anopheles gambiae*	XP_315650.4
Ag CHT-4	*Anopheles gambiae*	XP_315351.4
Ag CHT5-1	*Anopheles gambiae*	HQ456129.1
Ag CHT5-2	*Anopheles gambiae*	HQ456130.1
Ag CHT5-3	*Anopheles gambiae*	HQ456131.1
Ag CHT5-4	*Anopheles gambiae*	HQ456132.1
Ag CHT5-5	*Anopheles gambiae*	HQ456133.1
Ag CHT-7	*Anopheles gambiae*	XP_308858.4
Ag CHT-8	*Anopheles gambiae*	XP_316448.2
Ag CHT-9	*Anopheles gambiae*	XP_307732.4
Ag CHT-10	*Anopheles gambiae*	XP_001238192.2
Ag CHT-11	*Anopheles gambiae*	XP_310662.4
Ag CHT-12	*Anopheles gambiae*	XP_316142.4
Ag CHT-13	*Anopheles gambiae*	XP_314312.4
Ag CHT-16	*Anopheles gambiae*	XP_319801.4

Ag CHT-23	*Anopheles gambiae*	XP_001688641.1
Ag CHT-24	*Anopheles gambiae*	XP_316256.4
Ag CHT-IDGF2	*Anopheles gambiae*	XP_001237925.1
Ag CHT-IDGF4	*Anopheles gambiae*	XP_317398.3
Ag ZNT-1	*Anopheles gambiae*	XP_040161679.1
Ag ZNT-8	*Anopheles gambiae*	XP_313758.4
Ag ZNT-12	*Anopheles gambiae*	XP_061514025.1
Ag ZIP-9	*Anopheles gambiae*	XP_319959.4
Ag ZIP-10	*Anopheles gambiae*	XP_061511220.1
Ag ZIP-11	*Anopheles gambiae*	XP_061505917.1
Ag ZIP-13	*Anopheles gambiae*	XP_061498323.1
Ag mfrn	*Anopheles gambiae*	Q7Q7L5
Ag Ferritin-1	*Anopheles gambiae*	ABR88147.1
Ag Ferritin-2	*Anopheles gambiae*	ABR88146.1
Ag Cox17	*Anopheles gambiae*	XP_061512957.1
Ag ATP7	*Anopheles gambiae*	XP_061516829.1
Ae Nvd	*Aedes aegypti*	XP_001655171.1
Ae Cyp18a1	*Aedes aegypti*	EAT43716.1

Ae Did	*Aedes aegypti*	XP_001661673.2
Ae Shd	*Aedes aegypti*	AAX85208.1
Ae Fpps	*Aedes aegypti*	XP_001663796.1
Ae ALDH-1	*Aedes aegypti*	EAT33638.1
Ae ALDH-2	*Aedes aegypti*	EAT38124.1
Ae ALDH-3	*Aedes aegypti*	EAT39148.1
Ae ALDH-4	*Aedes aegypti*	EAT36141.1
Ae Jhamt	*Aedes aegypti*	XP_001651876.1
Ae JHE-1	*Aedes aegypti*	EAT43357.2
Ae JHE-2	*Aedes aegypti*	EAT43353.1
Ae JHE-3	*Aedes aegypti*	EAT43354.2
Ae JHE-4	*Aedes aegypti*	EAT32289.2
Ae JHE-5	*Aedes aegypti*	EAT34913.1
Ae JHE-6	*Aedes aegypti*	EAT39447.1
Ae JHE-7	*Aedes aegypti*	EAT43355.1
Ae JHE-8	*Aedes aegypti*	EAT43356.1
Ae JHE-9	*Aedes aegypti*	EAT44295.2
Ae JHEH-1	*Aedes aegypti*	AAM88326.1

Ae JHEH-2	*Aedes aegypti*	AAO52658.1
Ae JHEH-3	*Aedes aegypti*	ADZ76080.1
Ae Cht1	*Aedes aegypti*	EAT42806.1
Ae Cht2	*Aedes aegypti*	EAT35472.1
Ae Cht5	*Aedes aegypti*	XP_001656231.1
Ae Cht7	*Aedes aegypti*	EAT43684.1
Ae Cht10	*Aedes aegypti*	EAT35577.1
Ae Cht11	*Aedes aegypti*	EAT38324.1
Ae Cht17	*Aedes aegypti*	EAT39651.1
Ae Cht18	*Aedes aegypti*	EAT38348.1
Ae Cht20	*Aedes aegypti*	EAT45648.1
Ae IDGF2	*Aedes aegypti*	EAT46826.1
Ae IDGF4	*Aedes aegypti*	EAT46823.1
Ae ATP7	*Aedes aegypti*	XP_021709335.1
Hs ZNT-1	*Homo sapiens*	Q9Y6M5.3
Hs ZNT-2	*Homo sapiens*	CAI17131.1
Hs ZNT-3	*Homo sapiens*	Q99726.2
Hs ZNT-4	*Homo sapiens*	O14863.2

Hs ZNT-5	*Homo sapiens*	AAM09099.1
Hs ZNT-6	*Homo sapiens*	NP_060434.2
Hs ZNT-7	*Homo sapiens*	AAM21969.1
Hs ZNT-8	*Homo sapiens*	AAM80562.1
Hs ZNT-9	*Homo sapiens*	NP_006336.3
Hs ZNT-10	*Homo sapiens*	NP_061183.2
Hs ZIP-1	*Homo sapiens*	NP_055252.2
Hs ZIP-2	*Homo sapiens*	AAF35832.1
Hs ZIP-3	*Homo sapiens*	NP_653165.2
Hs ZIP-4	*Homo sapiens*	NP_570901.3
Hs ZIP-5	*Homo sapiens*	NP_775867.2
Hs ZIP-6	*Homo sapiens*	NP_036451.4
Hs ZIP-7	*Homo sapiens*	CAA20238.1
Hs ZIP-8	*Homo sapiens*	NP_071437.3
Hs ZIP-9	*Homo sapiens*	BAA92100.1
Hs ZIP-10	*Homo sapiens*	BAA86579.2
Hs ZIP-11	*Homo sapiens*	BAC04504.1
Hs ZIP-12	*Homo sapiens*	BAB70848.1

Hs ZIP-13	*Homo sapiens*	AAH08853.2
Hs ZIP-14	*Homo sapiens*	BAA06685.2
Hs NRAMP-1	*Homo sapiens*	P49279.1
Hs NRAMP-2	*Homo sapiens*	P49281.2
Hs Cox17	*Homo sapiens*	NP_001368931.1
Hs MTF-1	*Homo sapiens*	NP_005946.2
Mu ZNT-1	*Mus musculus*	Q60738.1
Mu ZNT-2	*Mus musculus*	NP_001034766.1
Mu ZNT-3	*Mus musculus*	NP_035903.2
Mu ZNT-4	*Mus musculus*	O35149.2
Mu ZNT-5	*Mus musculus*	AAL96438.1
Mu ZNT-6	*Mus musculus*	NP_659047.2
Mu ZNT-7	*Mus musculus*	NP_075703.1
Mu ZNT-8	*Mus musculus*	NP_766404.1
Mu ZNT-9	*Mus musculus*	AAV85854.1
Mu ZNT-10	*Mus musculus*	NP_001028458.1
Mu ZIP-1	*Mus musculus*	NP_038929.2
Mu ZIP-2	*Mus musculus*	NP_001034765.2

Mu ZIP-3	*Mus musculus*	NP_598896.3
Mu ZIP-4	*Mus musculus*	NP_082340.1
Mu ZIP-5	*Mus musculus*	NP_082368.1
Mu ZIP-6	*Mus musculus*	NP_631882.2
Mu ZIP-7	*Mus musculus*	AAC69903.1
Mu ZIP-8	*Mus musculus*	NP_080504.3
Mu ZIP-9	*Mus musculus*	XP_484158.3
Mu ZIP-10	*Mus musculus*	BAC65765.1
Mu ZIP-11	*Mus musculus*	BAC33713.1
Mu ZIP-12	*Mus musculus*	AAH89362.1
Mu ZIP-13	*Mus musculus*	NP_080997.1
Mu ZIP-14	*Mus musculus*	NP_659057.2
Mu Cox17	*Mus musculus*	BAB32486.1
Mu MTF-1	*Mus musculus*	CAA50470.1
CfJHE	*Choristoneura fumiferana*	AAD34172
TmJHE	*Tenebrio molitor*	AAL41023
HvJHE,	*Heliothis virescens*	AAC38822
BmJHE	*Bombyx mori*	AAR37335

mitoferrin-1	*Danio rerio*	NP_001035060.1
mitoferrin-2	*Danio rerio*	NP_998284.2
Mrs3p	*Saccharomyces cerevisiae*	CAY80651.2
Mrs4p	*Saccharomyces cerevisiae*	CAY81132.1
As Cyp18a1	*Anopheles sinensis*	KFB43110.1
