# Supplementary figures and images for: Genome Annotation of Molting-Related Protein-Coding Genes in Propsilocerus akamusi Reveals Transcriptomic Responses to Heavy Metal Contamination
Source: Insects. 2025 Jun 17;16(6):636. doi: 10.3390/insects16060636 (PMC12193260; doi:10.3390/insects16060636)

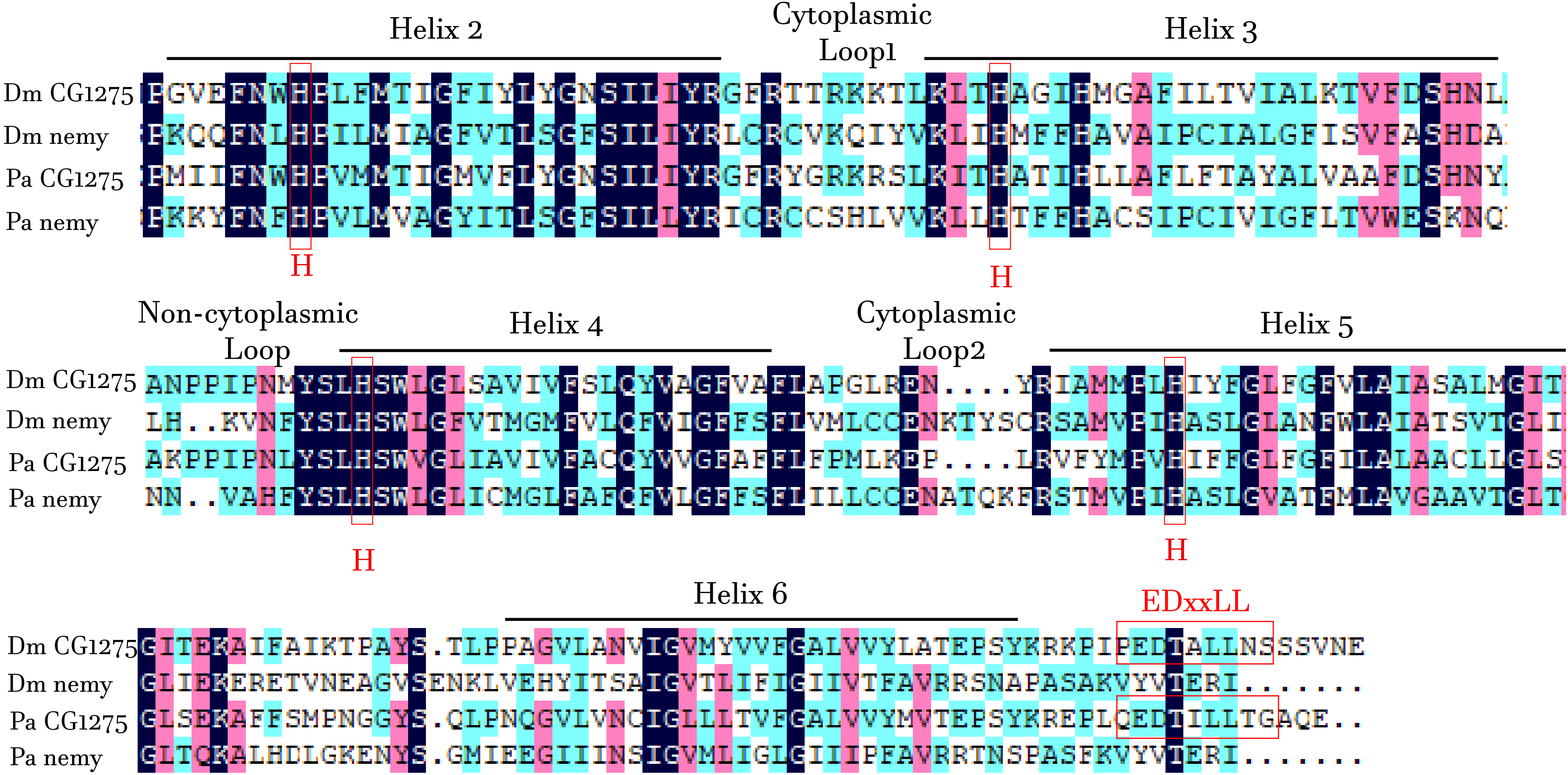

Supplement: Supplementary file 1 [file insects-16-00636-s001.zip › Figure S1.pdf]

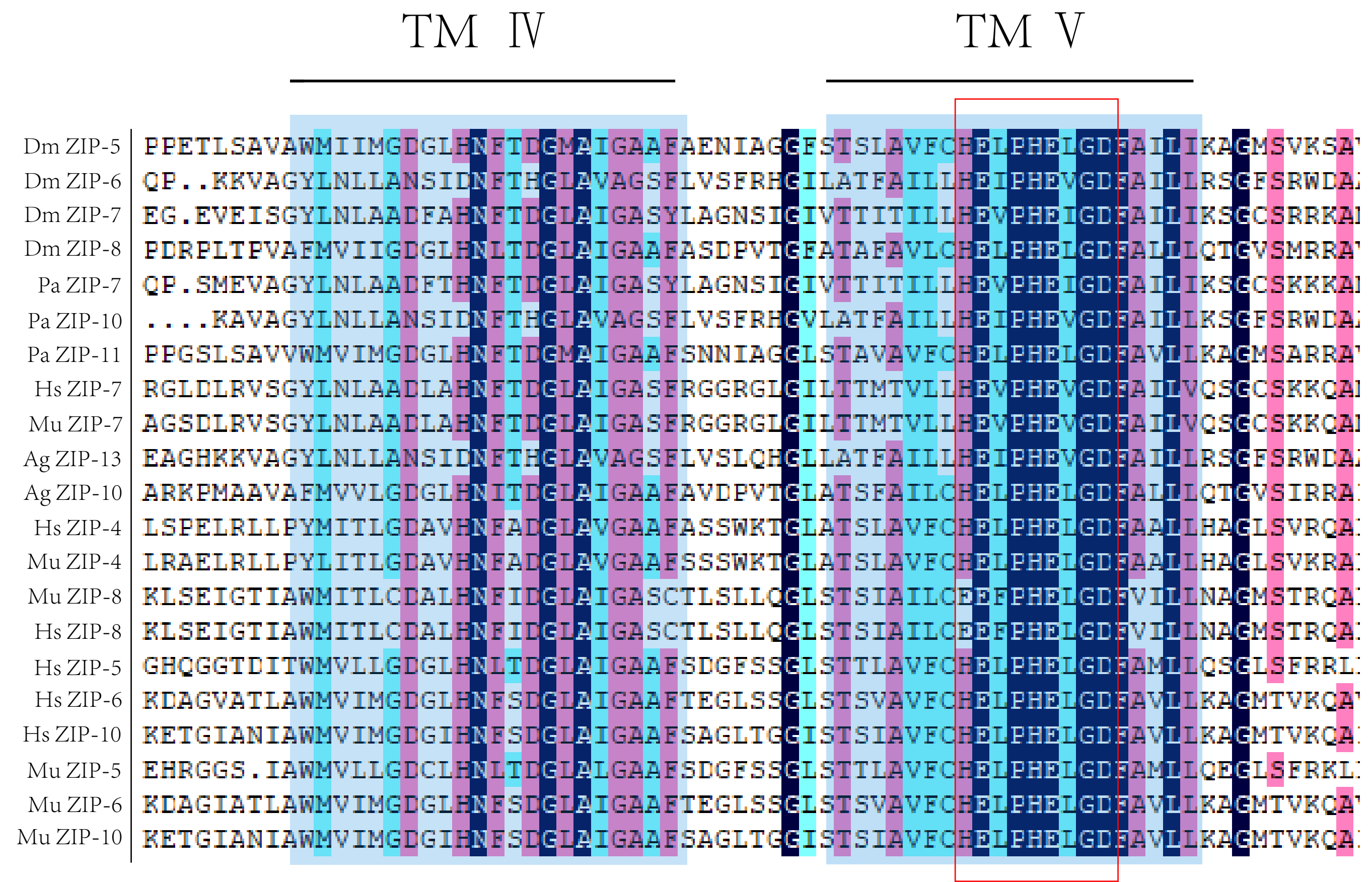

HE<sub>x</sub>PHE<sub>x</sub>GD

Supplement: Supplementary file 1 [file insects-16-00636-s001.zip › Figure S5.pdf]

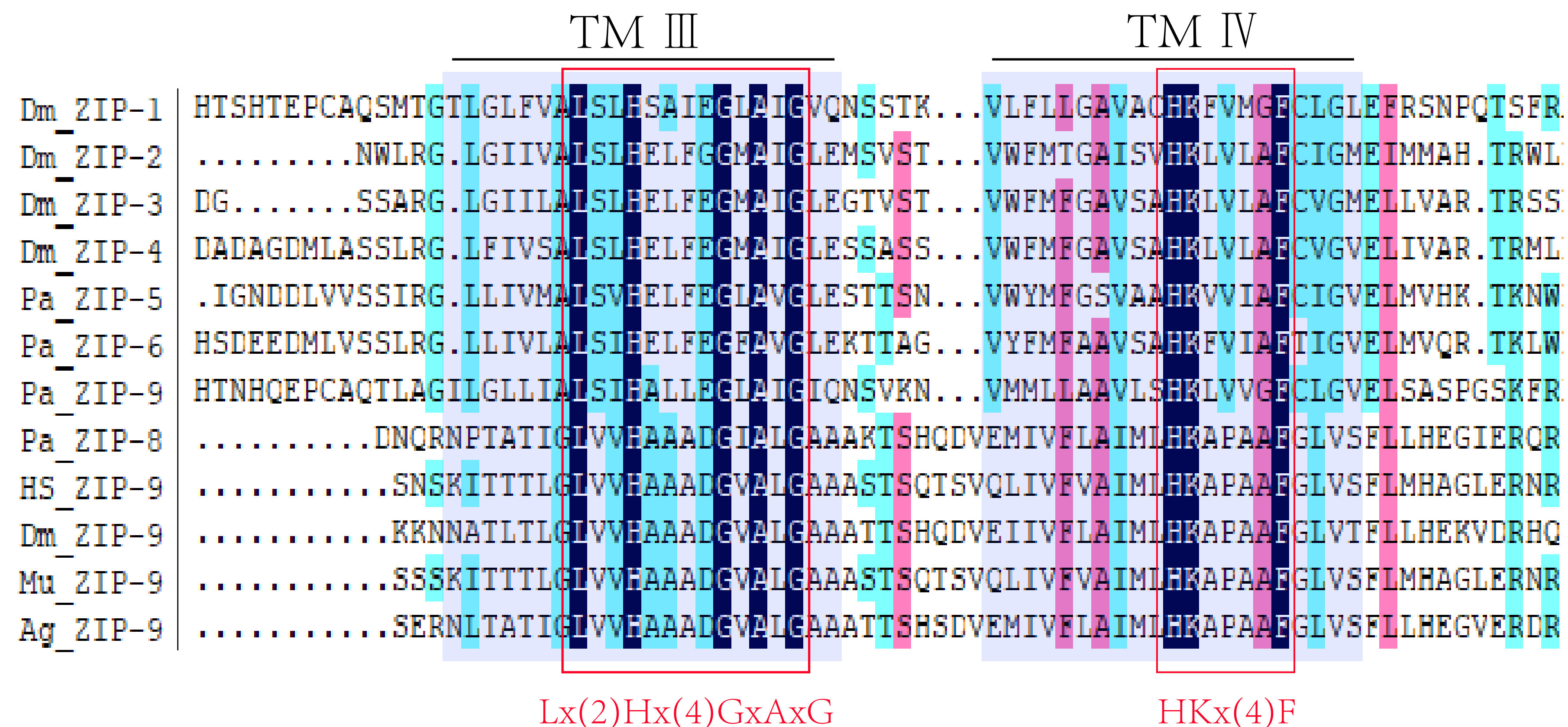

Supplement: Supplementary file 1 [file insects-16-00636-s001.zip › Figure S6.pdf]
